# ApoE deficiency protects from mRNA vaccine-induced mitochondrial dysfunction at the injection site under metabolic stress

**DOI:** 10.7150/thno.119545

**Published:** 2025-08-16

**Authors:** Sun-Hee Cho, Byeongkwon Choi, Jisun Lee, Yu-Sun Lee, Mi-Ock Baek, You-Jeung Lee, Chae-Ok Gil, Min-Kyung Choi, Sana Abdul Khaliq, Syeda Maham, Jae-Kyung Hyun, Gahyun Roh, Huijeong Choi, Sowon Lee, Seo-Hyeon Bae, Seonghyun Lee, Hyo-Jung Park, Jae-Hun Ahn, Na-Young Lee, Byeong-Cheol Kang, Young Kyo Seo, Byung-Kwan Lim, Jae-Hwan Nam, Mina Rho, Mee-Sup Yoon

**Affiliations:** 1 Department of Health Sciences and Technology, Gachon Advanced Institute for Health Science & Technology, Gachon University, Incheon, Republic of Korea.; 2 Department of Computer Science, Hanyang University, Seoul, Republic of Korea.; 3 Department of Medical and Biological Sciences, The Catholic University of Korea, Bucheon, Gyeonggi-do, Republic of Korea.; 4 Department of Biotechnology, The Catholic University of Korea, Bucheon, Gyeonggi-do, Republic of Korea.; 5 Lee Gil Ya Cancer and Diabetes Institute, Gachon University, Incheon, Republic of Korea.; 6 Department of Molecular Medicine, College of Medicine, Gachon University, Incheon 21999, Republic of Korea.; 7 Department of Biomedical Science, Jungwon University, Goesan-gun, Chungcheongbuk-do, Republic of Korea.; 8 Department of Experimental Animal Research, Biomedical Research Institute, Seoul National University Hospital, Seoul, Republic of Korea.; 9 Department of Veterinary Pathology and Research Institute of Veterinary Science, College of Veterinary Medicine, Seoul National University, Seoul, Republic of Korea.; 10 Graduate School of Translational Medicine, Seoul National University College of Medicine, Seoul, Republic of Korea.; 11 Aging Convergence Research Center, Korea Research Institute of Bioscience and Biotechnology (KRIBB), Daejeon, Republic of Korea.; 12 Department of Biomedical Informatics, Hanyang University, Seoul, Republic of Korea.

**Keywords:** mRNA vaccine, ApoE deficiency, mitochondrial dysfunction, injection site injury, immune response

## Abstract

**Rationale:** The local tissue effects of mRNA vaccination remain incompletely understood. We investigated how SARS-CoV-2 mRNA vaccination impacts injection site tissues in the context of different metabolic states and apolipoprotein E (ApoE) status.

**Methods:** We administered intramuscular SARS-CoV-2 mRNA vaccination to wild-type and ApoE-deficient mice under regular and high-fat diets, as well as macaques. We performed transcriptomic analysis, ultrastructural examination, functional assessments including grip strength testing, and immunological evaluations to characterize local and systemic responses.

**Results:** Intramuscular vaccination induced localized injury characterized by inflammation, mitochondrial disruption, and reduced grip strength in wild-type animals. Transcriptomic and ultrastructural analyses revealed denervation-associated changes, downregulation of mitochondrial genes, cristae disruption, and activation of immune and apoptotic pathways. ApoE-deficient mice, particularly under Western diet conditions, demonstrated protection from mitochondrial and inflammatory responses despite comparable vaccine expression levels. This protection involved attenuated mitochondrial gene downregulation, preserved mitochondrial DNA integrity, and reduced inflammatory responses. While ApoE deficiency resulted in diminished antibody production, T cell responses remained intact, indicating selective immunomodulation. Cardiac tissue showed minimal transcriptional changes, confirming injection site-specific effects.

**Conclusions:** ApoE deficiency provides protection against vaccine-induced mitochondrial and inflammatory damage at the injection site, with enhanced protective effects under metabolic stress conditions. These findings reveal important interactions between metabolic status, lipid metabolism, and local vaccine responses that may inform vaccination strategies in different patient populations.

## Introduction

mRNA vaccines have transformed modern vaccinology by offering a rapid, adaptable platform for combating infectious diseases 1. Their success during the COVID-19 pandemic—most notably with the Pfizer-BioNTech and Moderna vaccines—demonstrated their scalability and strong immunogenicity 2, 3. These vaccines deliver synthetic mRNA encapsulated in lipid nanoparticles (LNPs), enabling host cells to produce viral antigens that stimulate both innate and adaptive immune responses 4, 5. While generally well-tolerated, localized adverse events—such as muscle pain, swelling, and, in some cases, myositis—have raised concerns about muscle-specific side effects at the injection site 6-11. These symptoms, including reduced exercise tolerance and elevated serum creatine kinase levels, are indicative of substantial muscle tissue injury after vaccination 6-8. Despite being the primary target of vaccine administration, the injection site has received limited attention as a biologically active tissue. Understanding its role is essential not only for managing local reactogenicity but also for elucidating the broader mechanisms of vaccine-induced tissue remodeling.

Following intramuscular administration, antigen expression occurs not only in antigen-presenting cells but also in muscle fibers themselves, potentially triggering local inflammation 12, 13. Although mRNA and LNPs are typically degraded within 24 h, residual antigen may persist in tissue-resident immune cells, sustaining immune activation 14. This response recruits innate immune cells—including neutrophils, monocytes, and dendritic cells—to the injection site, where they, along with muscle cells, activate pattern recognition receptors, induce type I interferon signaling, trigger inflammasome activation, and enhance antigen presentation 5. While these coordinated processes contribute to the strong immunogenicity of mRNA vaccines, they may also disrupt muscle cellular homeostasis and promote tissue injury, particularly in individuals predisposed to inflammatory conditions 15.

Beyond direct immune mechanisms, host factors—such as age, sex, body composition, and metabolic health—can significantly influence vaccine outcomes 16-18. Among these, cholesterol metabolism has emerged as a key modulator of immune function and viral entry 19, 20, potentially affecting local responses to vaccination. Apolipoprotein E (ApoE), a central regulator of lipid transport and clearance via low-density lipoprotein (LDL) receptors, is essential for maintaining lipid homeostasis 21. ApoE-deficient mice, a widely used model of hypercholesterolemia, exhibit elevated systemic lipid levels and altered immune profiles 22, 23. However, it remains unclear how ApoE deficiency and the associated metabolic alterations impact injection site-specific responses to mRNA vaccination.

In this study, we investigated the effects of mRNA vaccination on muscle tissue at the injection site using both non-human primates (*Macaca fascicularis*) and mouse models (*Mus musculus*). By combining physiological, histological, and transcriptomic analyses, we characterized muscle responses under normal and hyperlipidemic conditions, including wild-type and ApoE-deficient mice fed a regular chow or Western diet. Our findings reveal that mRNA vaccination induces mitochondrial dysfunction, inflammation, and structural disruption at the injection site, accompanied by transcriptional changes reminiscent of denervation. To our knowledge, this is the first study to demonstrate that ApoE deficiency, particularly under a Western diet, attenuated vaccine-induced muscular alterations, providing protection against mitochondrial dysfunction and inflammatory responses at the injection site. Taken together, our findings establish the injection site as a biologically dynamic and metabolically sensitive microenvironment that critically influences local responses to mRNA vaccination.

## Methods

### Preparation of mRNA

The antigen was designed using the DNA sequence encoding the spike protein of the SARS-CoV-2 Omicron variant. To construct the mRNA vaccine plasmid, the antigen DNA was inserted into multiple cloning sites on the mRNA platform using restriction enzymes Pac1 and Cla1, as previously described 24. The plasmid was then linearized with Not1 to prepare the mRNA template. mRNA was synthesized using the EZ T7 High Yield *In vitro* Transcription Kit (Enzynomics, Daejeon, Korea) according to the manufacturer's protocol. The capping reaction was performed using SC101 (STPharm, Siheung, Korea), and uridine triphosphate (UTP) was substituted with N1-methyl-pseudouridine (Trilink, San Diego, CA, USA). The resulting mRNA was precipitated using lithium chloride and subsequently purified using cellulose, following established procedures 25.

### Lipid nanoparticle formulation for the mRNA vaccine

Lipid nanoparticles (LNPs) were prepared according to a reported protocol 26. Briefly, all lipid components were dissolved in ethanol at a molar ratio of 50:10:38.5:1.5 (SM-102, distearoylphosphatidylcholine, cholesterol, and 1,2-dimyristoyl-rac-glycero-3-methoxypolyethylene glycol-2000, respectively), and mRNAs were dissolved in sodium citrate buffer (50 mM; pH 4) solution at a charge ratio of N/P = 6. LNPs were formulated using NanoAssemblr® IgniteTM (Precision Nanosystems, Vancouver, Canada) by mixing aqueous and organic solutions at a ratio of 3:1 and a total flow rate of 10 mL/min. The LNP solution was concentrated by ultrafiltration using an Amicon Ultracentrifugal filter (UFC9030; Merck Millipore, Billerica, MA, USA) following the manufacturer's instructions.

### Nonhuman primate model and vaccination protocol

Four-to five-year-old cynomolgus macaque (*Macaca fascicularis*) monkeys were purchased from ORIENT GENIA (Seongnam, Gyeonggi-do, South Korea). Animals were maintained at the Nonhuman Primate Research Center of Seoul National University Hospital under controlled conditions (20-28 ℃, 30-70% humidity, 12 h light/dark cycle). Animals were fed a Teklad Global 20% Protein Primate Diet (Inotiv, Cat#: 2050c, West Lafayette, IN, USA) and fresh fruits. All animal experimental procedures were approved by the Institutional Animal Care and Use Committee of Seoul National University Hospital (Approval No. 23-0029). Animals were injected intramuscularly (left brachialis muscle) with either SARS-CoV-2 mRNA vaccine (mRNA dosage, 800 µg/head) or normal saline as a control. The vaccination schedule involved administering the primary immunization, followed by three additional booster immunizations at 2-week intervals. The animals were sacrificed 2 days after the final immunization, and the injection site (left brachialis muscle) tissue, particularly the visible lesion, was collected, followed by cut into ~1 cm^3^ pieces for formalin fixation or RNAlater^TM^ (Invitrogen, Cat#: AM7021, Waltham, MA, USA).

### Mice model and vaccination protocol

Female ApoE^tm1Unc^ and wild-type C57BL/6J mice were obtained from Jackson Laboratory (Bar Harbor, ME, USA). The mice were housed in a controlled environment, maintained at a constant temperature of 22°C, with a 12 h light/dark cycle throughout the experiment period. To establish a Western Diet (WD), 6-week-old mice were fed a diet containing 0.2% cholesterol and 42% fat (Cat#: DY-88137, Doo Yeol Biotech, Seoul, Korea). A separate group of 6-week-old mice was provided a regular chow diet (RCD) for 10 weeks. All experimental procedures were reviewed and approved by the Institutional Animal Care and Use Committee (IACUC) and conducted in accordance with their guidelines (permission number: LCDI-2022-0070). Mice on either the WD or RCD were intramuscularly injected with the SARS-CoV-2 mRNA vaccine or normal saline as a control. The vaccination schedule consisted of a primary immunization administered at 14 weeks of age, followed by a booster immunization at 16 weeks of age. Two days after the booster immunization, the mice were euthanized, and tissue and blood samples were collected for subsequent analysis.

### RNA sequencing and data processing

Paired-end sequencing reads were generated using the Illumina Sequencing NovaSeq platform. Prior to analysis, Trimmomatic v0.39 27 was employed to eliminate adapter sequences and trim low-quality bases. The preprocessed reads were then aligned to the *Mus musculus* (GRCm39) and *Macaca fascicularis* (macFas5) reference genomes using HISAT v2.2.1 28. The reference genome sequences and gene annotations were obtained from the NCBI Genome Assembly and NCBI RefSeq databases, respectively. Before alignment, the SAM files were sorted and indexed using SAMtools v1.9 29. After alignment, transcript assembly and quantification were performed using StringTie v2.2.1 30. Gene expression levels were quantified in terms of raw read counts, FPKM (Fragments Per Kilobase of transcript per Million mapped reads), and TPM (Transcripts Per Million).

### Differentially expressed gene analysis

For *Mus musculus* on a Western diet (WMM), genes with a zero count in any of the 20 samples were excluded, leaving 14,243 genes out of 40,879 for analysis. Similarly, for *Mus musculus* fed a regular chow diet (RMM), this filtering resulted in 14,349 genes of 40,879. For *Macaca fascicularis* fed a regular chow diet (RMF), 15,665 genes out of 28,832 were retained for analysis after filtering across six samples. The Wald test was conducted using DESeq2 v1.40.1 31, which fits a generalized linear model (GLM) for each gene and obtains p-values to identify differentially expressed genes (DEGs). DEGs is determined if they exhibited an absolute log2FoldChange ≥ 1 and a p-value < 0.01, with comparisons designating the first group as the case and the second as the control. The groups were defined as follows: Pre-vaccine ApoE^+/+^, Post-vaccine ApoE^+/+^, Pre-vaccine ApoE^-/-^, and Post-vaccine ApoE^-/-^ mice. To reduce sample variability for genes with low expression, the rlog transformation method was employed. This transformation converted count data to a log_2_ scale and normalized them using the library size factor, enhancing data visualization in the figures, particularly when significant differences in library size factors existed among samples. To facilitate standardized comparisons across different genes or conditions, the stat value—defined as the log2FoldChange divided by its standard error (lfcSE)—was used. This metric provided a more precise comparative analysis than log2FoldChange alone and was incorporated into the figures to improve interpretability.

### Data visualization and principal component analysis

Principal Component Analysis (PCA) was conducted to provide an overview of the data using R package Factoextra v1.0.7, focusing on either the intersection (887 genes) or union (1,125 genes) of DEGs across the samples. Hierarchical clustering heatmaps were generated using rlog-transformed expression values to visualize gene expression across all samples, including WMM, RMM, and RMF. Heatmaps were constructed with the 887 intersecting DEGs and applied z-score normalization to adjust for large differences in expression between genes. To account for interspecies variation, normalization was performed separately for WMM, RMM, and RMF. Hierarchical clustering was applied to the genes in x-axis using Euclidean distance and complete linkage, with dendrograms added for detailed visualization. Box plots were created to depict the distribution of normalized gene expression values across groups. Each box plot included the median value for each group, and p-values were indicated with asterisks (*) above the box plots to denote statistically significant differences between groups.

### KEGG pathway and functional analysis

KEGG pathway network analysis was performed using ClusterProfiler v4.8.3 32 to identify significant pathways (*p* < 0.001) within each comparison group. Pathways are visualized as nodes in a network graph, with edges representing the number of shared genes between pathways. The KEGG pathway visualization emphasized critical biological processes, including immune system responses, mitochondrial activity, apoptosis, metabolism, muscle structure, and cardiolipin synthase. Relationships and fold-change information for each gene were displayed within the network. Fold-change values derived from DESeq2stat were used to color the nodes, providing insight into the magnitude of gene expression changes. Connections between genes were illustrated as solid or dashed arrows, indicating direct or indirect interactions, respectively. Triangular arrows represented activation, while circular arrows indicated inhibition, offering a detailed view of the functional interactions and regulatory mechanisms within the identified pathways.

### Quantification of antigen-specific total IgG using enzyme-linked immunosorbent assay (ELISA)

Antigen-specific total IgG levels in mouse serum were quantified using enzyme-linked immunosorbent assays (ELISAs). Briefly, 96-well plates were coated with the S protein from the SARS-CoV-2 Omicron variant at a concentration of 100 ng/well and incubated overnight at 4°C. The wells were then blocked with 100 µl of blocking buffer (1% BSA in PBS) for 1 h at room temperature. Diluted serum samples were then added to the wells and incubated for 2 h at room temperature. Following the incubation, the wells were washed thrice with 200 µl of PBS-T (PBS containing Tween 20) to remove unbound components. A horseradish peroxidase (HRP) -conjugated anti-mouse IgG antibody (Bethyl Laboratories, Montgomery, TX, USA), diluted 1:5000 in 1% BSA buffer, was then added to each well and incubated for 1 h at room temperature. After three additional washes with PBS-T, tetramethylbenzidine (TMB) substrate was added, and the plates were incubated for 15 min. The reaction was terminated by adding 2 N H_2_SO_4_, and the optical density (OD) was measured at 450 nm using a microplate reader (GloMax Explorer, Promega, Seoul, Republic of Korea).

### Detection of IFN-γ secretion using enzyme-linked immunospot (ELISpot) assay

Splenocytes (5 × 10^5^ cells) isolated from immunized mice were cultured in 96-well MultiScreen-IP Filter Plates (Millipore, Burlington, MA, USA). The cells were stimulated with spike protein antigen peptides derived from the Wuhan SARS-CoV-2 strain (5 μg/mL) in RPMI medium and incubated for 24 h at 37°C. The secretion of IFN-γ by splenocytes was assessed using an ELISpot assay, performed according to the manufacturer's protocol (Mab-tech, Stockholm, Sweden).

### Flow cytometric analysis

To evaluate cytotoxic T cell responses, isolated splenocytes (1 × 10^6^ cells) were stimulated with a 5 μg/well peptide mixture from SARS-CoV-2 S protein (WNSNNLD, YQAGSTPCNGV, NCYFPLQSYGF, KNKCVNFNFNGLTGTGVLT, VFQTRAGCLIGAEHV) and treated with Brefeldin A (GolgiPlug; BD Biosciences, Franklin Lakes, NJ, USA) in RPMI medium for 12 h at 37°C. Splenocytes were then incubated with anti-mouse CD16/32 (Invitrogen, Waltham, MA, USA) for 20 min at 4°C to block Fc receptors. For surface staining, cells were treated with Fixable Viability Dye eFluor 520 (eBioscience, San Diego, CA, USA) and anti-mouse CD8a (clone 53-6.7, BioLegend, San Diego, CA, USA) fluorescent antibodies for 30 min at 4°C in the dark. Subsequently, cells were fixed and permeabilized using BD Cytofix/Cytoperm™ (BD Biosciences), followed by intracellular staining with anti-mouse IFN-γ (clone XMG 1.2; BioLegend) fluorescent antibodies for 30 min under same conditions. To assess effector memory T cells (T_EM_) and B cells, splenocytes were cultured in 96-well plates and incubated with CD16/32 (Invitrogen) for 20 min at 4°C. Surface staining was performed with Fixable Viability Dye eFluor™ 520 (eBioscience) and the following antibodies: CD4 (clone GK1.5; BioLegend), CD8 (clone 53-6.7; BioLegend), CD44 (clone IM7; BioLegend), CD62L (clone MEL-14; BioLegend), and CD19 (clone 6D5; BioLegend). The cells were fixed with 4% paraformaldehyde. For dendritic cell (DCs) and macrophage analyses, splenocytes from immunized mice were digested in RPMI medium containing 1 mg/mL collagenase D and 0.5 M EDTA (Sigma Aldrich, Burlington, MA, USA) for 15 min at 37 °C. The cells were stained with anti-mouse CD11c, F4/80, CD80, and MHC II antibodies using the same staining protocol. Flow cytometric analysis was performed using a CytoFlex flow cytometer (Beckman Coulter, Brea, CA, USA) and the data were analyzed using CytExpert software (Beckman Coulter).

### Histological and immunohistochemical analysis of muscle tissue

Muscle tissue at the injection site of the vaccine was fixed in 10% neutral formaldehyde, embedded in paraffin, and sectioned for hematoxylin and eosin (H&E) staining. To evaluate muscle damage, stained areas were scanned using the Motic EasyScan Digital Slide Scanner (Motic Hong Kong Ltd., Hong Kong, China), and 5-10 randomly selected fields containing centrally nucleated myofibers were imaged. Damaged muscle fibers were identified based on established histopathological criteria, including pale cytoplasm, reduced fiber diameter, angular morphology, central nucleation, and signs of cellular infiltration 33. The proportion of damaged fibers was calculated as a percentage of the total myofibers. To evaluate vascular remodeling, vessel structures were manually outlined and quantified using ImageJ software (NIH, Bethesda, MD, USA). The vascular area was calculated as the total vessel lumen area normalized to the total tissue area. All analyses were performed on at least five randomly selected fields per sample by investigators blinded to group identity.

### Immunohistochemistry

#### DAB-based Immunohistochemistry

Antigen retrieval was performed using Dako Retrieval Solution (pH 6.0, S2369; Dako, Santa Clara, CA, USA). Non-specific binding was blocked using Dako Protein Block Serum-Free (X0909; Dako) for 1 h at room temperature. Sections were then incubated overnight at 4 °C with anti-SARS-CoV/SARS-CoV-2 spike protein antibody (1:200; GTX632604, GeneTex, Irvine, CA, USA). Antigen detection was performed using the Dako Envision Detection System Peroxidase/DAB+ (K5007), and sections were counterstained with hematoxylin. The stained slides were dehydrated, mounted, and imaged using the Motic Easyscan Digital Slide Scanner (Motic Hong Kong, Ltd.).

#### Fluorescent Immunohistochemistry

Quadriceps muscle samples were collected 2 days after the second intramuscular injection of SARS-CoV-2 mRNA vaccine or saline. Tissues were embedded in OCT compound and snap-frozen in isopentane cooled in liquid nitrogen. Cryosections (10 μm) were fixed in 10% formalin for 15 min at room temperature, permeabilized in PBS with 0.1% Triton X-100 for 15 min, and blocked with 5% bovine serum albumin for 2 h. Primary antibodies were applied overnight at 4°C in blocking buffer. The following primary antibodies were used: anti-laminin (L9393, Sigma-Aldrich, Burlington, MA, USA), anti-PAX7 (PAX7, DSHB, Iowa City, IA, USA), and anti-Ki67 (ab15580, Abcam, Cambridge, UK). After PBS washes, sections were incubated with fluorescently labeled secondary antibodies for 1 h at room temperature: Alexa Fluor 488-conjugated anti-rabbit IgG (A11034, Invitrogen), Alexa Fluor 594-conjugated anti-mouse IgG (Z25007, Invitrogen), Alexa Fluor 555-conjugated anti-goat IgG (150078, Abcam), and FITC-conjugated anti-mouse IgM (F9259, Sigma-Aldrich). Nuclei were counterstained with DAPI (4′,6-diamidino-2-phenylindole), and coverslips were mounted using anti-fade mounting medium. Fluorescent images were acquired using a CKX3-HOUN microscope (Olympus, Tokyo, Japan) under identical imaging settings across all groups. Quantification of PAX7⁺/Ki67⁺ nuclei and IgM⁺ myofibers was performed on at least four randomly selected fields per sample using ImageJ software.

### Succinate dehydrogenase (SDH) Histochemistry

To assess mitochondrial oxidative enzyme activity, 10 μm cryosections of quadriceps muscle were incubated at 37°C for 2 h in SDH reaction buffer containing 0.2 M phosphate buffer (pH 7.4), 1.0 mg/mL nitro blue tetrazolium (NBT), and 0.1 M sodium succinate. Following incubation, slides were fixed in 10% formalin for 15 min, rinsed in distilled water, dehydrated through graded ethanol, and mounted with Permount. Stained sections were imaged using the Motic EasyScan Digital Slide Scanner (Motic Hong Kong Ltd., Hong Kong, China). Quantification of SDH⁺ myofibers was performed using ImageJ software (NIH, Bethesda, MD, USA), applying a fixed intensity threshold of 158 was applied to define positive regions.

### Transmission electron microscopy (TEM) and quantification of mitochondrial subtypes

Quadriceps muscles were harvested 2 days after the second intramuscular injection of SARS-CoV-2 mRNA vaccine or control buffer. Tissues were immediately fixed in 2% glutaraldehyde and 2% paraformaldehyde in 0.1 M phosphate buffer (pH 7.4) at 4°C overnight. Samples were post-fixed in 1% osmium tetroxide for 2 h, dehydrated through a graded ethanol series, and embedded in Poly/Bed 812 resin. Ultrathin sections (~80 nm) were prepared using an ultramicrotome (Leica EM UC7, Leica Microsystems) and stained with uranyl acetate and lead citrate. Images were acquired with a HT7800 transmission electron microscope (HITACHI, Tokyo, Japan) operated at 100 kV at the Electron Microscopy Core Facility of Yonsei University College of Medicine. Digital micrographs were captured using a side-mounted RC camera. Mitochondrial quantification was performed on longitudinal sections. Intermyofibrillar (IMF) and subsarcolemmal (SS) mitochondria were classified based on their anatomical location—between myofibrils and beneath the sarcolemma, respectively. Aberrant mitochondria were defined as those exhibiting morphological abnormalities such as swelling and disrupted cristae. For each sample, at least ten randomly selected fields were analyzed by a blinded observer, and mitochondria were manually categorized as either normal or aberrant. The number and proportion of each subtype were quantified using ImageJ software (NIH, Bethesda, MD, USA).

### Measurement of grip strength

Grip strength was assessed using a grip strength meter (Scitech, Seoul, Korea). Each mouse was positioned to grasp a horizontal bar connected to a force gauge, which recorded the maximum pull force exerted by the forelimbs prior to grip loss. After confirming a stable grip involving both forepaws, the force gauge was reset. The mouse was then gently pulled backward by its tail to elicit a forceful response against the bar. The peak force generated during the response was recorded by the gauge. The maximum grip force of each mouse was measured at least four times to ensure the accuracy and consistency. The recorded grip strength values were normalized to the body weight of the mice for comparative analysis.

### Analysis of mitochondrial copy number

Mitochondrial copy number was quantified by qRT-PCR, measuring the relative abundance of NADH dehydrogenase 1 (*ND1*), a mitochondrial gene, and hexokinase 2 (*HK2*), a nuclear gene. Total DNA was extracted from quadriceps tissues. Reactions were performed with TOPrealTM qPCR 2× PreMIX (SYBR Green with high ROX) kit (Enzynomics) under standard cycling conditions. The primer sequences were as follows: *ND1* forward, 5'- CTAGCAGAAACAAACCGGGC-3' and reverse, 5'-CCGGCTGCGTATTCTACGTT-3'; *HK2* forward, 5'-GCCAGCCTCTCCTGATTTTAGTGT-3' and reverse, 5'-GGGAACACAAAAGACCTCTTCTGG-3'. The relative mtDNA copy number was calculated using the 2^-ΔCt method, where ΔCt represents the Ct difference between *ND1* and *HK2*. Data were analyzed in triplicate and expressed as mean (standard deviation).

### Chronic inflammation model via subcutaneous lipopolysaccharide (LPS) infusion

Female C57BL/6 mice (6-8 weeks old; Dae-Han Biolink, Korea) were maintained under standard housing conditions with *ad libitum* access to food and water. All procedures were approved by the IACUC of the Samsung Biomedical Research Institute (Approval No. 2022032201), which is AAALAC-accredited and follows ARRIVE and ILAR guidelines. Mice were fed a standard diet (5% fat) and implanted subcutaneously with osmotic pumps (Alzet model 1004; DURECT Corp.) containing either Tween-saline (0.9% NaCl + 0.1% Tween-80), or LPS (300 μg/kg/day; *E. coli*, Sigma) diluted in Tween-saline. Pumps were implanted following manufacturer instructions. At the endpoint (2 days after the second mRNA vaccination), mice were anesthetized with isoflurane, and blood was collected via facial vein puncture. Mice were euthanized by CO₂ inhalation, and tissues were collected for analysis.

### Statistical analysis

Statistical analyses were conducted using GraphPad Prism software (version 9.0, GraphPad Software, La Jolla, CA, USA). Group comparisons were performed using a two-tailed parametric and nonparametric Mann-Whitney U test or one-way ANOVA, depending on the dataset. Results are presented as mean (standard deviation). A P-value of less than 0.05 was considered statistically significant, with significance levels denoted as *p < 0.05, **p < 0.01, and ***p < 0.001.

## Results

### mRNA vaccination-induced damage at the injection site in non-human primate models

**Figure 1:**
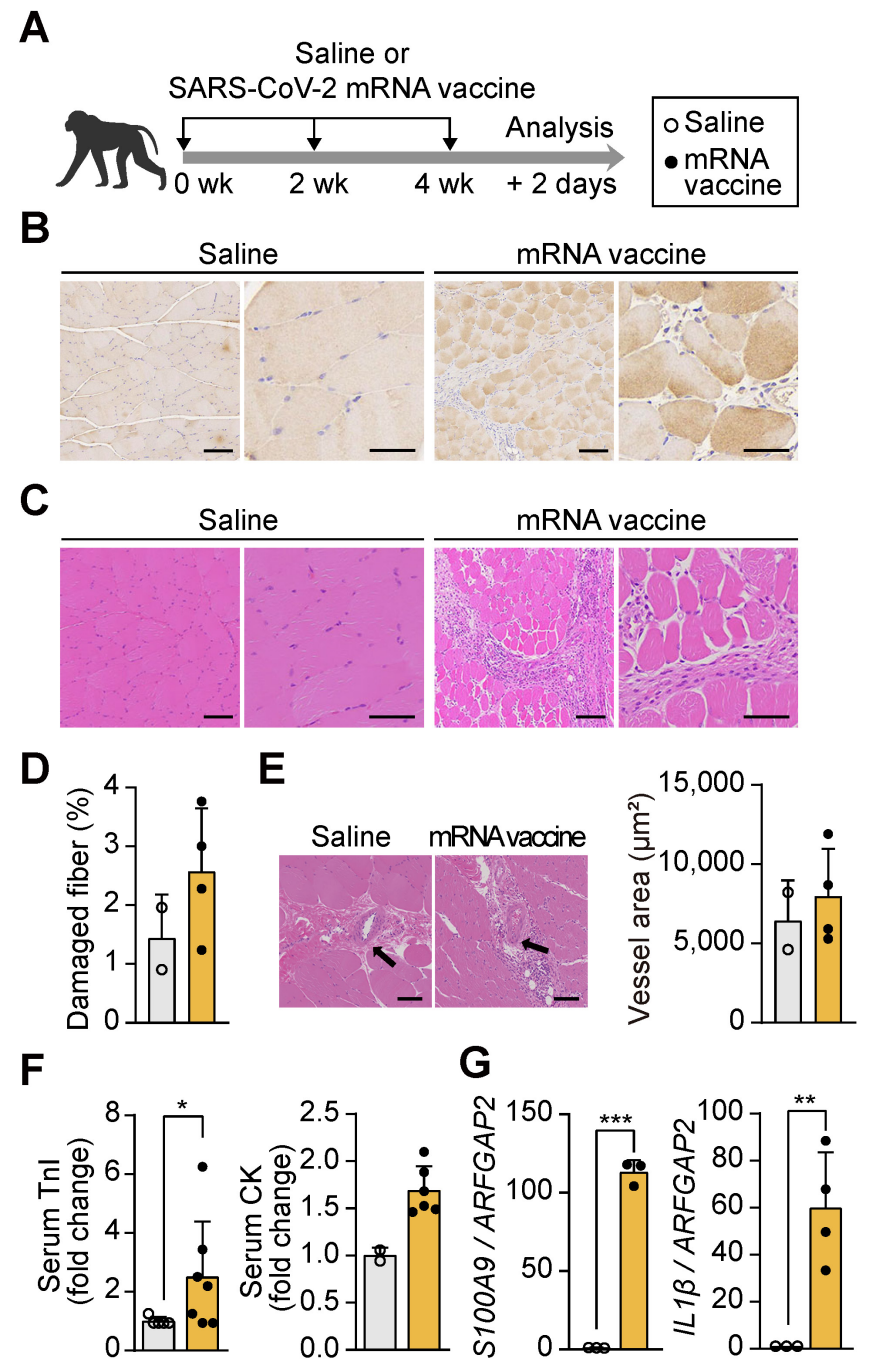
** mRNA vaccination induces localized tissue damage at the injection site in non-human primates and mice.** (A) Schematic of the vaccination protocol. Macaca fascicularis received three intramuscular doses of SARS-CoV-2 mRNA vaccine at 14-day intervals and were sacrificed 48 h after the final dose. (B) Immunohistochemical detection of SARS-CoV-2 spike protein in quadriceps muscle using an anti-SARS-CoV-2 spike antibody. Scale bars: 100 μm (left), 60 μm (right). (C) Hematoxylin and eosin (H&E) staining of vaccinated quadriceps showing features of muscle injury. Scale bars: 100 μm (left), 60 μm (right). (D) Quantification of damaged myofibers based on established histological criteria, normalized to total myofiber count using ImageJ (n = 2, saline; n = 4, mRNA). (E) Quantification of vascular area in quadriceps sections using ImageJ. Scale bars: 100 μm (n = 2, saline; n = 4, mRNA). (F) Serum troponin I (TnI) level (n = 5, saline; n = 7, mRNA) and creatine kinase (CK) activity (n = 2, saline; n = 5, mRNA) measured 48 h after the final injection. (G) Relative expression of S100A9 and IL1β mRNA in quadriceps measured by qRT-PCR and normalized to ARFGAP2 (n = 3, saline; n = 3-4 mRNA). Data are presented as mean (standard deviation). Statistical significance was determined using a two-tailed Student's t-test (*p < 0.05, **p < 0.01, and ***p < 0.001).

To investigate the effects of mRNA vaccination on muscle tissue at the injection site, cynomolgus macaques (*Macaca fascicularis*) received three doses of a SARS-CoV-2 mRNA vaccine at 14-day intervals (Figure 1A), and injection site muscle tissue was collected 48 h after the final dose. The mRNA vaccine, encoding the spike protein of the SARS-CoV-2 Omicron variant, was designed to target the region between the 5' and 3' untranslated regions (UTRs) (Figure S1A). Encapsulation in lipid nanoparticles (LNPs), with an average particle size (Z-average) of 108.2 nm and a zeta potential of 9.32 mV, ensured stability and efficient delivery of the mRNA, maintaining a stable physicochemical profile essential for optimal vaccine performance 34 (Figure S1B-C). Immunohistochemical analysis confirmed the presence of the SARS-CoV-2 spike protein at the injection site using anti-SARS-CoV-2 spike protein antibodies (Figure 1B). Histological examination revealed significant morphological alterations in the vaccinated muscle, including myofiber degeneration, reduced fiber diameter, pale cytoplasm, angular fiber shapes, central nuclei, and cellular infiltration, as observed through hematoxylin and eosin (H&E) staining (Figure 1C and 1D). These pathological changes align with the established criteria for muscle injury 33, characterized by myofiber degeneration and the release of intracellular components that activate inflammatory cascades 35, 36. However, vascularization around the muscle fibers, essential for nutrient delivery during muscle regeneration, remained unchanged (Figure 1E). Serum troponin I (TnI) levels and creatine kinase (CK) activity, both key biomarkers of muscle damage 37, 38, were elevated by more than 2.5- and 1.5-fold, respectively, in vaccinated macaques compared to controls (Figure 1F). In parallel, the expression of pro-inflammatory genes *S100A9* and interleukin-1 β (*IL1β*), which are central to inflammatory modulation 39, was significantly upregulated in muscle tissue following vaccination (Figure 1G). These results indicate that acute inflammatory and structural damage at the injection site in non-human primates, without impairing vascular architecture.

### Injection site injury and functional impairment in mRNA vaccinated mice

**Figure 2:**
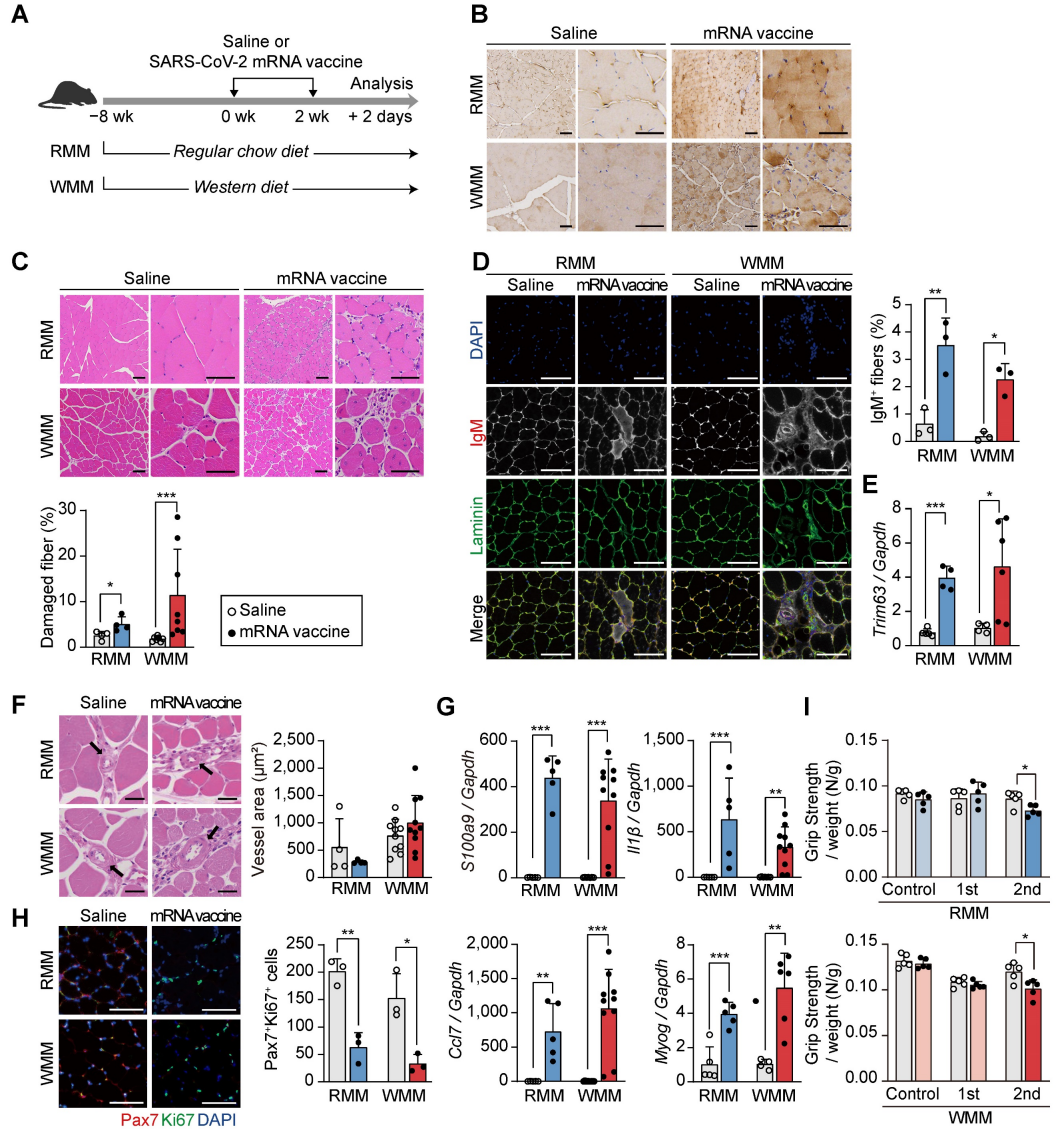
** Intramuscular mRNA vaccination induces muscle damage in mice fed a regular chow diet or a Western diet.** (A) Schematic of the experimental design. Mice were fed either a regular chow diet (RCD; RMM) or a Western diet (WD; WMM) for 8 weeks, followed by two intramuscular injections of SARS-CoV-2 mRNA vaccine at 2-week intervals. Mice were sacrificed 48 h after the second dose. (B) Spike protein expression in quadriceps muscle was detected by immunohistochemistry using an anti-SARS-CoV-2 spike antibody. Scale bars: 100 μm (left), 60 μm (right). (C) Hematoxylin and eosin (H&E) staining and quantification of damaged fiber. Damaged myofibers were identified based on established histological criteria and quantified using ImageJ. Scale bars: 100 μm (left), 60 μm (right) (n = 4, RMM; n = 7-8, WMM). (D) Representative immunofluorescence images and quantification of IgM⁺ myofibers. DAPI (blue), laminin (green), and IgM (originally red) were visualized, with IgM rendered in grayscale to improve contrast. Scale bar: 100 μm (n = 3, RMM; n = 3, WMM). (E) Expression of Trim63 mRNA, normalized to Gapdh (n = 5, RMM; n = 4-6, WMM). (F) Vascular area in quadriceps muscle was measured using ImageJ. Scale bars: 40 μm (n = 4, RMM; n = 10, WMM). (G) Relative expression levels of S100a9, Il1β, Ccl7, and Myog in quadriceps, normalized to Gapdh (n = 5, RMM; n = 4-10, WMM). (H) Representative images and quantification of PAX7⁺/Ki67⁺ nuclei by immunofluorescence. Scale bar: 100 μm (n = 3, RMM; n = 3, WMM). (I) Grip strength was measured 24 h before vaccination and 24 h after the first and second doses. Measurements were repeated at least four times per mouse and normalized to body weight (n = 5, RMM; n = 5, WMM). Data are represented as mean (standard deviation). Statistical significance was determined using a two-tailed Student's t-test; *p < 0.05, **p < 0.01, and ***p < 0.001.

To further evaluate the effects of mRNA vaccination at the injection site, we administered two intramuscular doses of the vaccine into the quadriceps of mice, spaced two weeks apart, and collected muscle tissue 48 h after the second injection (Figure 2A). Mice were maintained on an RCD or a Western diet (WD) for eight weeks, allowing a comparative assessment of vaccine responses under different metabolic conditions. The groups were designated as RMM (Regular chow-fed *Mus musculus*) and WMM (Western diet-fed *Mus musculus*) (Figure 2A). Spike protein expression was confirmed at the injection site by immunohistochemistry (Figure 2B). H&E staining revealed increased numbers of injured myofibers in vaccinated mice compared to controls (Figure 2C). This was further supported by a rise in IgM-positive myofibers, a marker of muscle injury (Figure 2D). Additionally, expression of* Trim63*, a muscle-specific E3 ubiquitin ligase involved in protein degradation, was elevated in both dietary groups (Figure 2E).

Despite these indicators of muscle damage, vascularization surrounding muscle fibers remained unchanged (Figure 2F), implying preserved perfusion. Inflammatory responses were also evident, as vaccinated mice exhibited increased expression of *S100a9* and *Il1β* in muscle tissue under both dietary conditions (Figure 2G). Moreover, *Ccl7*, a chemokine linked to Cxcl5^+^ fibroblasts and associated with muscle injury 40, 41, was significantly upregulated (Figure 2G). *Myog*, a key marker of muscle differentiation, was also upregulated in both RMM and WMM groups, suggesting an active muscle repair response (Figure 2G). However, the number of PAX7^+^ Ki67^+^ satellite cells, indicative of actively proliferating muscle stem cells in skeletal muscle, was significantly reduced, suggesting impaired regenerative capacity (Figure 2H). Functionally, vaccinated mice displayed a significant decline in grip strength following the second dose compared to saline-injected controls, independent of dietary condition (Figure 2I). These findings demonstrate that mRNA vaccination induces injection site injury characterized by myofiber degeneration, inflammation, and reduced functional capacity, regardless of metabolic state.

### Transcriptomic alterations at the injection site following mRNA vaccination

**Figure 3:**
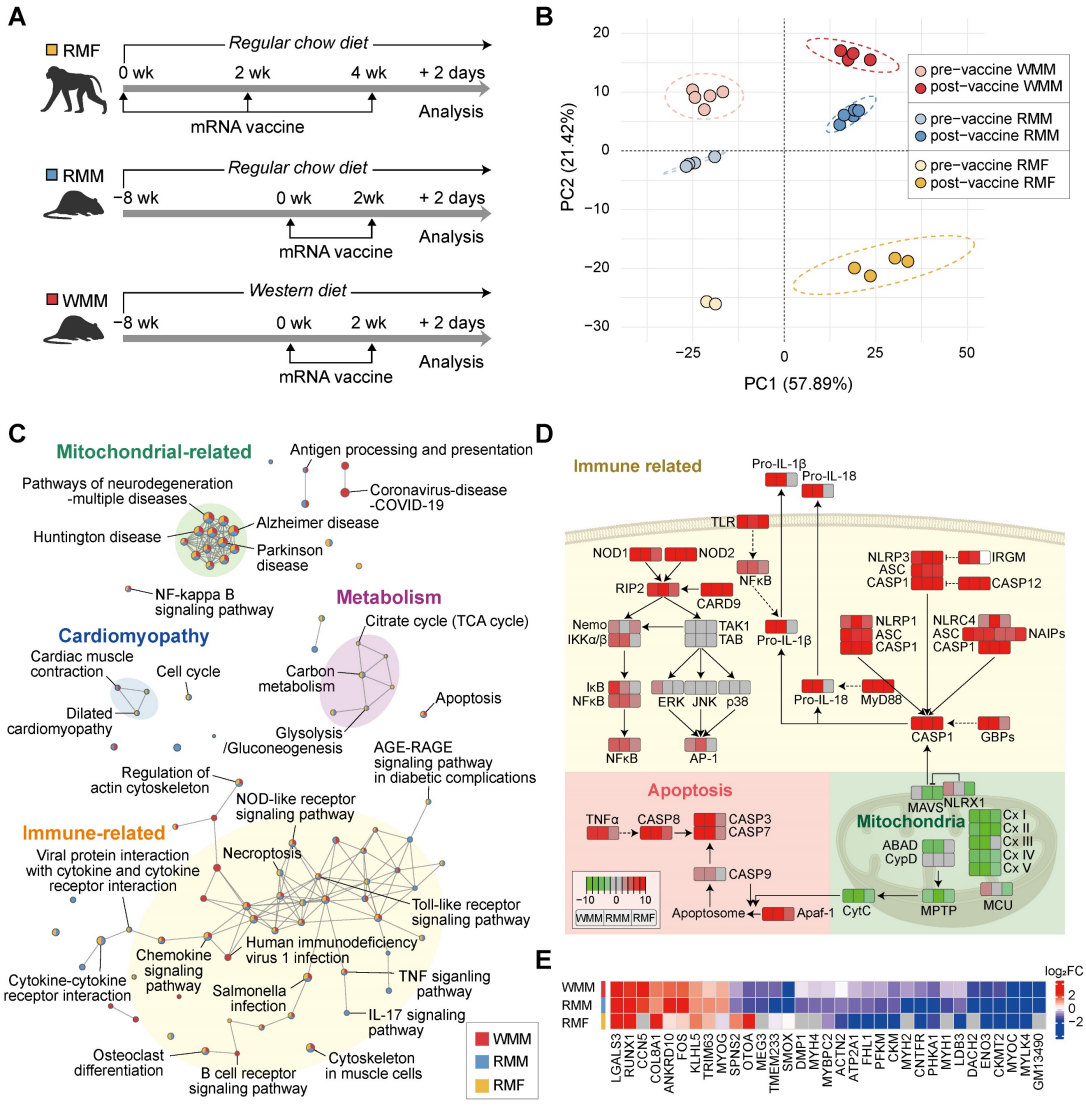
** mRNA vaccination induces coordinated transcriptional responses at the injection site.** (A) Overview of the experimental design (see also Figure 1A and Figure 2A). Macaca fascicularis fed a regular chow diet (RMF) received three mRNA vaccine doses at 14-day intervals and were sacrificed 2 days after the final injection. Mice on either a regular chow diet (RCD; RMM) or a Western diet (WD; WMM) received two vaccine doses and were sacrificed 48 h after the second injection. (B) Principal component analysis (PCA) of pre- and post-vaccination transcriptomes in RMF, RMM, and WMM groups. Only pathways with p < 0.01 were included. (C) Enrichment map showing KEGG pathways across groups; nodes represent significantly enriched pathways (p < 0.01), edges indicate shared DEGs. Pathways are color-coded by category: immune (yellow), mitochondrial (green), cardiomyopathy-related (blue), and metabolic (violet). (D) Pathway analysis showing upregulated immune and apoptosis genes, and downregulated mitochondrial electron transport chain (ETC) genes. In KEGG annotations, pro- and cleaved caspases are annotated separately; in our dataset, transcripts for CASP1 and CASP9 were grouped due to shared identifiers. (E)

Building on our initial observations of tissue injury at the injection site following mRNA vaccination, we conducted transcriptomic analysis to evaluate broader gene expression changes in both non-human primates and mice. We analyzed differentially expressed genes (DEGs) before and after vaccination in three groups: RCD-fed *Macaca fascicularis* (RMF), RCD-fed *Mus musculus* (RMM), and WD-fed *Mus musculus* (WMM) (Figure 3A). A total of 887 DEGs were identified across all three groups, revealing both common and unique DEGs among the groups as visualized by Venn diagram (Figure S2A).

Hierarchical clustering of DEG expression profiles showed that gene expression changes between pre- and post-vaccine samples were more substantial than differences among species or diet groups (Figure S2B). This trend confirmed by principal component analysis (PCA), where vaccination status accounted for the largest proportion of variance across samples (Figure 3B), suggesting a shared core transcriptional response to mRNA vaccination. To investigate biological processes affected by vaccination, we performed pathway enrichment analysis and identified two major categories of changes (Figure 3C): (1) upregulated immune and apoptotic signaling pathways, and (2) downregulated mitochondrial activity pathways. Additionally, cardiomyopathy-related (blue circle) and metabolic (violet circle) pathways showed shared expression patterns in both RMM and RMF groups, although these changes were less pronounced in the RMF group. The immune/apoptotic response included robust upregulation of caspase family members such as *CASP1, CASP3, CASP7*, and *CASP8*, which are key executors of inflammatory and programmed cell death responses (Figure 3D). These transcriptomic signatures were supported by histological evidence, with increased TUNEL-positive nuclei indicating elevated apoptosis in muscle tissue across all groups (Figure S2C). In parallel, genes involved in mitochondrial function were markedly suppressed post-vaccination. This included downregulation of immunometabolic regulators such as Mitochondrial Antiviral Signaling Protein (*MAVS*) and NLR family member X1 (*NLRX1*), as well as widespread repression of genes encoding components of all five oxidative phosphorylation (OXPHOS) complexes (Figure 3D). To explore potential mechanistic parallels between mRNA vaccine-induced tissue injury and denervation, we compared transcriptional changes with datasets from denervated muscle 42-44. This comparison was motivated by the downregulation of structural and mitochondrial genes—features commonly observed in denervated muscle—which may reflect underlying bioenergetic and structural instability. This analysis revealed 33 overlapping DEGs, including genes associated with extracellular organization (*COL8A1, LGALS3*), muscle and neuron differentiation (*FOS, MYOG*), and cytoskeletal structure (*LDB3, ACTN2, CKM, ENO3, FHL1, MYBPC2, MYH1, MYH4, MYH2*) (Figure 3E). Notably, many of these shared genes showed similar directionality in expression, suggesting a partial convergence of vaccine-induced and denervation-associated transcriptional responses. Together, these findings demonstrate that mRNA vaccination induces coordinated transcriptional remodeling at the injection site, characterized by activation of immune-apoptotic pathways and suppression of mitochondrial genes, partially recapitulating features of denervation-induced muscle remodeling.

### Mitochondrial impairment at the injection site following mRNA vaccination

**Figure 4:**
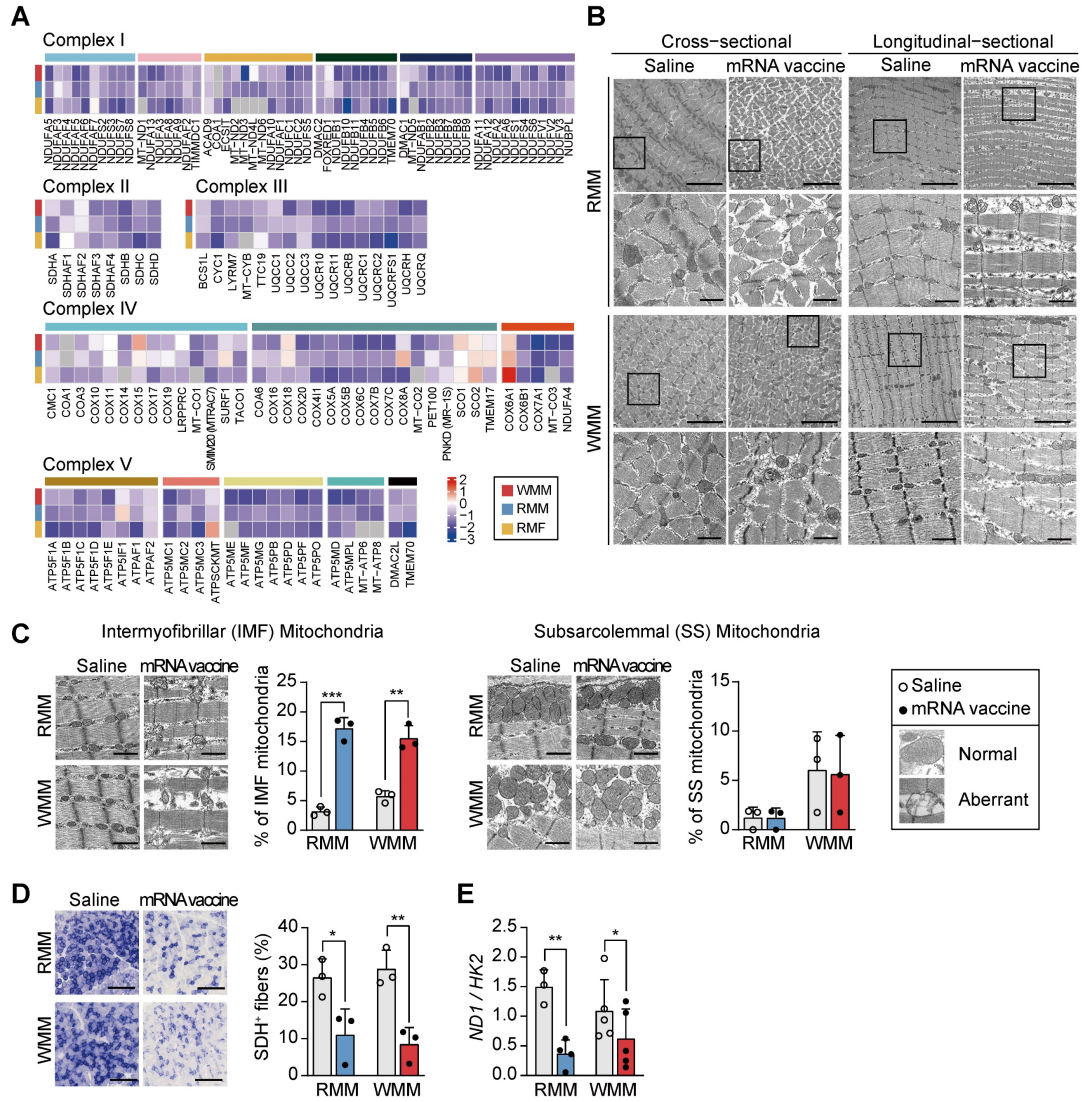
** mRNA vaccination disrupts mitochondrial architecture and function at the injection site.** (A) Heatmap showing fold changes in differentially expressed genes (DEGs) related to mitochondrial complexes I-V across RMF, RMM, and WMM groups, demonstrating consistent post-vaccination downregulation. (B) Transmission electron microscopy (TEM) images of quadriceps muscle in cross-sectional and longitudinal views showing mitochondrial ultrastructural changes. The boxed regions in each micrograph indicate the areas that are shown at higher magnification directly below the corresponding images. Scale bars: 5.0 μm (top), 1.0 μm (bottom). (C) Quantification of morphologically normal and abnormal mitochondria in intermyofibrillar (IMF) and subsarcolemmal (SS) regions using ImageJ. Scale bar: 1.0 μm (n = 3, RMM; n = 3, WMM). (D) Representative SDH-stained cross-sections of quadriceps muscle and quantification of SDH⁺ myofibers using ImageJ. Scale bar: 300 μm (n = 3, RMM; n = 3, WMM). (E) Mitochondrial DNA content assessed by the ratio of NADH dehydrogenase 1 (ND1) to hexokinase 2 (HK2) via qPCR (n = 3-4, RMM; n = 5, WMM). Data are presented as mean (standard deviation). Statistical significance was assessed by a two-tailed Student's t-test (*p < 0.05, **p < 0.01, and ***p < 0.001).

Our transcriptomic analysis revealed that mRNA vaccination significantly impairs mitochondrial function at the injection site. Genes involved in glycolysis, pyruvate dehydrogenase activity, and the TCA cycle were consistently downregulated (Figure S3A), alongside reduced expression of major structural muscle proteins-including troponin C (TnC: TNNC1, TNNC2), tropomyosin (TPM1, TPM2), skeletal muscle actin (ACTA1), and myosin heavy chains and light chains (MYH family) (Figure S3B). Notably, these transcriptional patterns mirrored those seen in our denervation comparison, with suppression of cytoskeletal genes such as ACTN2 and myosin isoforms, suggesting overlap in molecular response (Figure S3B). Genes associated with mitochondrial respiratory complexes I-V were downregulated across all groups post-vaccination (Figure 4A). Additionally, reduced expression of PTPMT1 and CRLS1, essential for cardiolipin synthesis, suggested compromised mitochondrial membrane integrity (Figure S3C). Upregulation of genes in the glycerol-3-phosphate to phosphatidyl-glycerophosphate (PGP) pathway may reflect a compensatory response to the reduced cardiolipin production (Figure S3C). To determine whether these transcriptomic changes were accompanied by structural alterations, we performed transmission electron microscopy (TEM). In mRNA-vaccinated muscle tissue, longitudinal sections revealed Z-band disruption and disorganized myofibrillar architecture, accompanied by swollen intermyofibrillar (IMF) mitochondria with cristae damage. Cross-sectional views showed irregular sarcomere arrangement and prominent mitochondrial enlargement, indicating widespread ultrastructural injury (Figure 4B). Notably, the number of aberrant IMF mitochondria—exhibiting swelling and disrupted cristae—increased post-vaccination, while subsarcolemmal (SS) mitochondria remained largely unaffected (Figure 4C). This differential susceptibility suggests spatial heterogeneity in vaccine-induced mitochondrial responses, potentially reflecting the distinct functional roles of IMF mitochondria in contractile function versus SS mitochondria in membrane-associated processes 45, 46. The selective impairment of IMF mitochondria, coupled with Z-band disruption, further supports a mechanistic connection between structural disorganization and impaired bioenergetics in the affected muscle tissue.

Furthermore, the intensity of succinate dehydrogenase (SDH; mitochondrial complex II) staining was markedly reduced in mRNA vaccine-injected site, indicating diminished mitochondrial enzymatic activity (Figure 4D). Mitochondrial DNA copy number also significantly decreased in both RMM and WMM groups, consistent with TEM images (Figure 4E). Collectively, these results demonstrate that mRNA vaccination causes mitochondrial impairment at the injection site, characterized by selective damage to IMF mitochondria, reduced enzymatic activity, and loss of mitochondrial content.

### ApoE deficiency protects against injection site injury following mRNA vaccination

**Figure 5:**
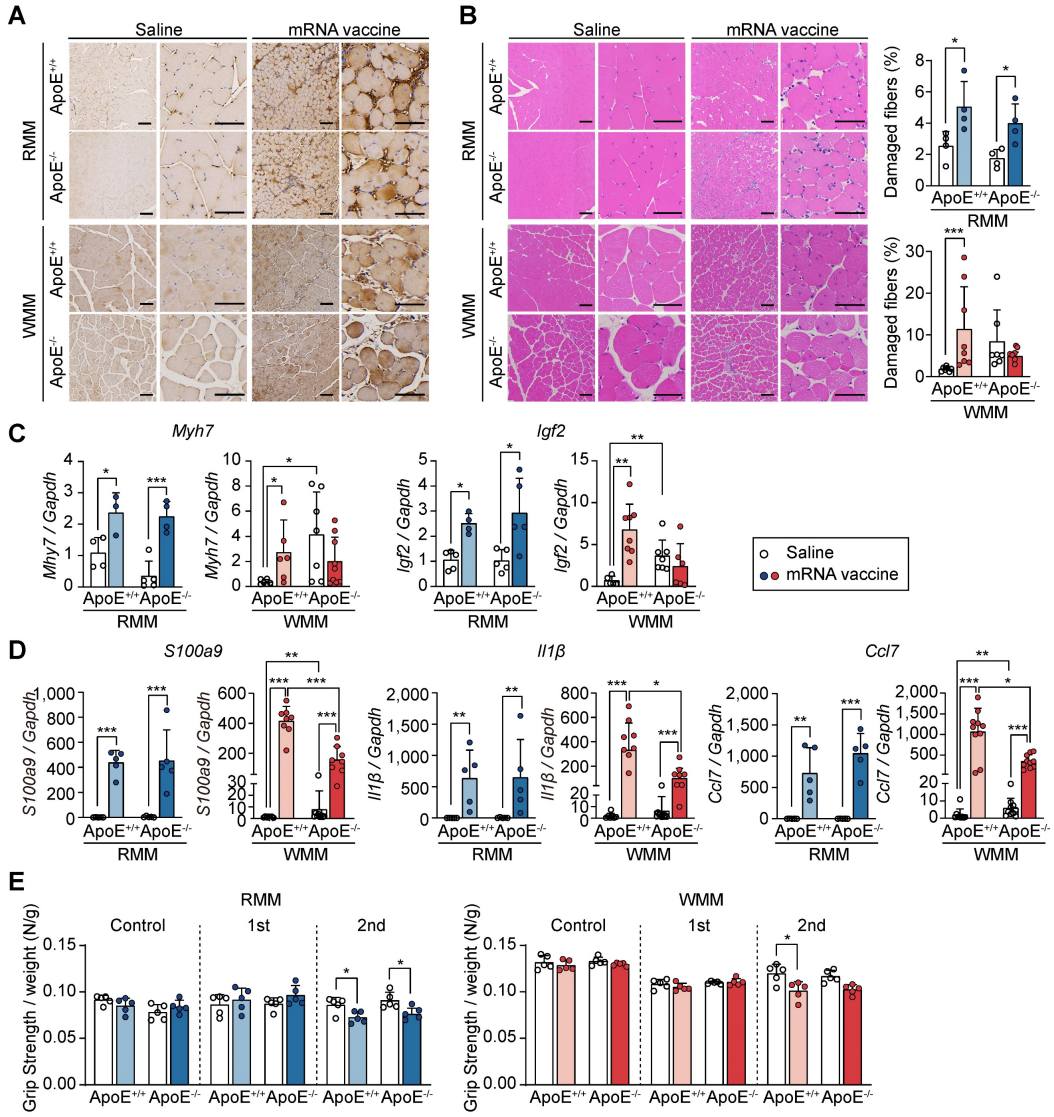
** ApoE deficiency under Western diet attenuates injection site injury following mRNA vaccination.** Mice were fed either a regular chow diet (RCD) or a Western diet (WD) for 8 weeks and received two mRNA vaccine doses at 2-week intervals. Quadriceps tissue was collected 48 h after the final injection. (A) Immunohistochemical detection of SARS-CoV-2 spike protein in quadriceps. Scale bars: 100 μm (left), 60 μm (right). (B) Hematoxylin and eosin (H&E) staining and quantification of damaged myofibers using established histological criteria. Scale bars: 100 μm (left), 60 μm (right) (n = 4, RMM; n = 7-8, WMM). (C) Expression of Myh7 and Igf2 at the injection site, measured by qRT-PCR and normalized to Gapdh (n = 3-5, RMM; n = 5-9, WMM). (D) Quantification of S100a9, Il1β, and Ccl7 mRNA expression by qRT-PCR, normalized to Gapdh (n = 5, RMM; n = 7-10, WMM). (E) Grip strength measured at baseline and 24 h after the first and second vaccine doses (n = 5, RMM; n = 5, WMM). Data are shown as mean (standard deviation). Statistical significance was determined by a two-tailed Student's t-test (*p < 0.05, **p < 0.01, and ***p < 0.001).

Following our observation of injection site injury in macaque monkeys and mice under RCD and WD conditions, we sought to evaluate how ApoE deficiency modifies this response. Given the role of cholesterol in facilitating viral entry and modulating immune responses 47, we employed ApoE^-/-^ mice, a model of hypercholesterolemia 47, to investigate how cholesterol imbalance affects mRNA vaccine-induced responses at the injection site. To model hypercholesterolemia, ApoE^+/+^ and ApoE^-/-^ mice were placed on either RCD or WD for 8 weeks. Differences in body size and weight were observed across groups, with ApoE^-/-^ mice showing increased body weight, particularly under WD conditions (Figure S4A-B). As expected, plasma total cholesterol and triglyceride levels were markedly elevated in ApoE^-/-^ mice, confirming the successful induction of hypercholesterolemia (Figure S4C). Before evaluating tissue responses to mRNA vaccination, we first confirmed that ApoE deficiency does not alter mRNA delivery or antigen expression. To this end, we intramuscularly administered LNP-encapsulated mRNA-luciferase and measured luciferase activity after D-luciferin injection. At 3 h post D-luciferin injection, both ApoE^+/+^ and ApoE^-/-^ mice exhibited similar luciferase expression at the injection site (Figure S5A). Biodistribution analysis up to 24 h revealed similar expression profiles across tissues between genotypes (Figure S5B), indicating that ApoE deficiency does not alter mRNA vaccine delivery or expression. We next assessed local tissue damage following vaccination. Quadriceps and other tissues were collected 48 h after the second mRNA vaccine dose (Figure S6A). While organ size remained largely unchanged, splenomegaly was observed in WD-fed ApoE^-/-^ mice (Figure S6B). Immunohistochemical staining of injected sites confirmed spike protein expression across all groups (Figure 5A).

Histologically, ApoE^+/+^ mice showed increased muscle fiber damage regardless of diet, and a similar trend was seen in RCD-fed ApoE^-/-^ mice. However, in WD-fed ApoE^-/-^ mice, the number of damaged fibers did not significantly increase following vaccination (Figure 5B). Vessel area was unchanged across all conditions, indicating preserved vascular support (Figure S6C). To explore the molecular basis underlying these histological differences, we examined the expression of muscle differentiation and inflammatory markers. Gene expression analysis showed that *Myh7* and *Igf2*, muscle differentiation markers, were upregulated post-vaccination in ApoE^+/+^ and RCD-fed ApoE^-/-^ mice, but remained low in WD-fed ApoE^-/-^ mice (Figure 5C). Additionally, mRNA levels of inflammatory cytokines *S100a9*, *Il1β*, and *Ccl7* were upregulated in response to mRNA vaccination in ApoE^+/+^ and RCD-fed ApoE^-/-^ mice (Figure 5D), whereas they were significantly attenuated in WD-fed ApoE^-/-^ mice (Figure 5D), indicating blunted inflammatory signaling in this context. To determine whether these molecular and histological changes were reflected in functional outcomes, we next assessed muscle strength. Grip strength was reduced in ApoE^+/+^ mice following vaccination regardless of the dietary condition and similarly declined in RCD-fed ApoE^-/-^ mice. In contrast, WD-fed ApoE^-/-^ mice exhibited preserved grip strength post-vaccination consistent with a protective effect at the injection site (Figure 5E). These findings suggest that ApoE deficiency, especially in the context of a WD, attenuates injected site injury and inflammation following mRNA vaccine administration, without affecting antigen delivery.

### ApoE gene status alters immune responses to mRNA vaccination in mice

**Figure 6:**
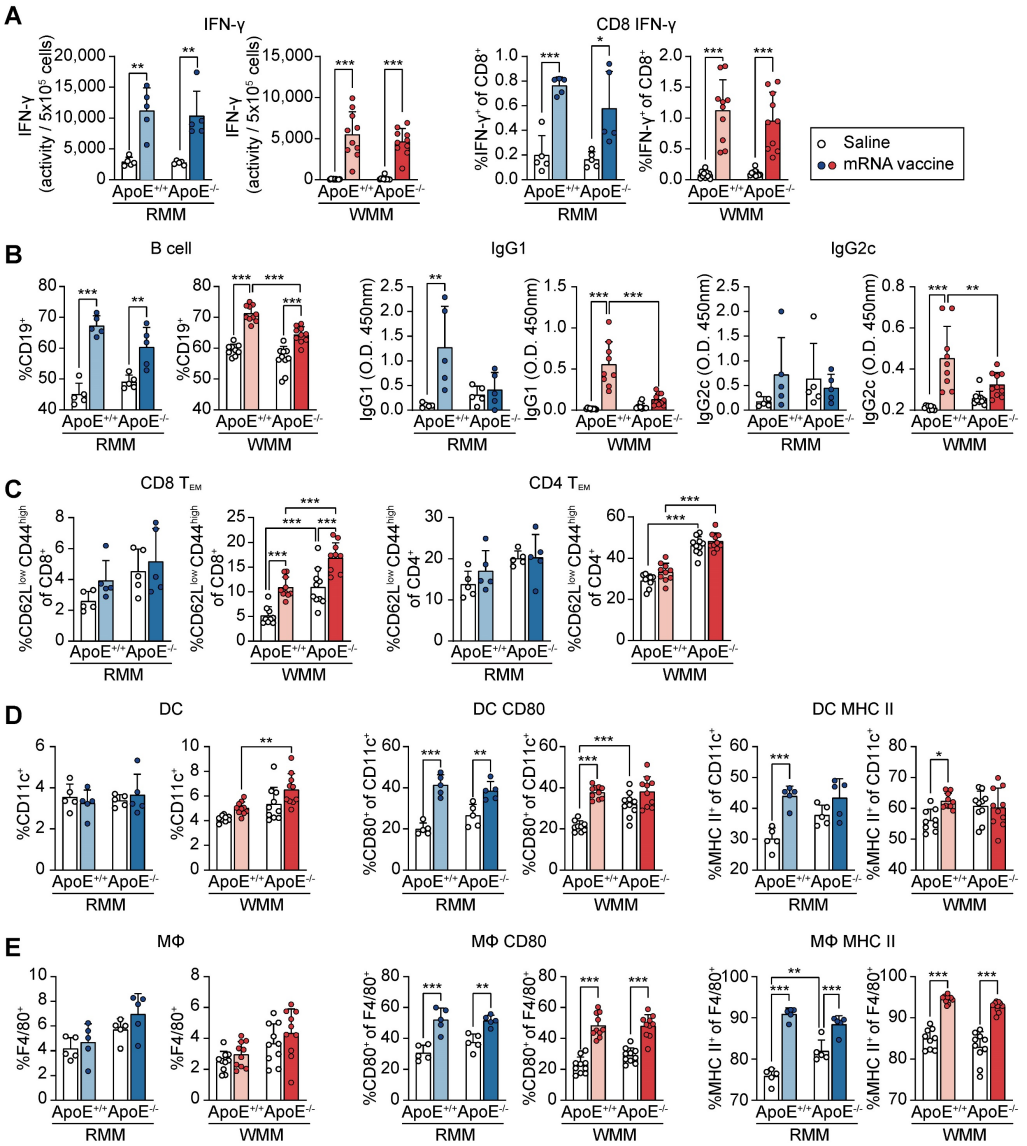
** Immune profiling of ApoE+/+ and ApoE-/- mice following mRNA vaccination.** All analyses were performed in ApoE+/+ and ApoE-/- mice fed a regular chow diet (RCD) or Western diet (WD), following intramuscular administration of SARS-CoV-2 mRNA vaccine or saline control. (A) Interferon-γ (IFN-γ) production was quantified by ELISA and normalized per 5 × 10⁵ cells. CD8⁺ T cell-specific IFN-γ responses were assessed by ELISpot and flow cytometry (FACS), reported as the percentage of IFN-γ⁺ CD8⁺ cells (n = 5, RMM; n = 10, WMM). (B) B cell populations were quantified by flow cytometry as the percentage of CD19⁺ cells. Serum IgG1 and IgG2c antibody levels were measured by ELISA (n = 5, RMM; n = 9-10, WMM). (C) Effector memory T cells (Tᴇᴍ) subsets were analyzed by FACS (CD62Llow CD44high) (n = 5, RMM; n = 9-10, WMM). (D) Dendritic cells (DCs) (CD11c⁺) were analyzed for expression of CD80 and MHC II (n = 5, RMM; n = 10, WMM). (E) Macrophage (MΦ) (F4/80⁺) activation markers CD80 and MHC II were quantified (n = 5, RMM; n = 9-10, WMM). Statistical analysis was performed using one-way ANOVA. *p < 0.05, **p < 0.01, and ***p < 0.001.

To assess the efficacy of mRNA vaccination within the context of hypercholesterolemia, we evaluated variations in immune responses across different diets and their interactions with ApoE gene status. Mice were grouped and maintained on either an RCD or a WD for eight weeks. Each group then received either the mRNA vaccine or saline injections at two-week intervals, with evaluations conducted 48 h after the second injection (Figure S6A). We first examined cellular immune responses by assessing IFN-γ-secreting cells and IFN-γ production by CD8^+^ T-cells (Figure 6A). mRNA vaccination significantly enhanced both measures in ApoE^+/+^ and ApoE^-/-^ mice under RCD and WD conditions, with comparable fold changes between genotypes. These results suggest that ApoE deficiency does not markedly affect cellular immune responses to mRNA vaccination.

Next, we assessed humoral immunity. While vaccination increased B cell frequencies (CD19⁺) in most groups, the resulting frequencies were significantly lower in WD-fed ApoE^-/-^ mice than in their ApoE^+/+^ counterparts (Figure 6B). IgG1 levels rose significantly in ApoE^+/+^ mice under both diets, while no significant change was observed in ApoE^-/-^ mice. Similarly, IgG2c levels increased following vaccination in WD-fed ApoE^+/+^ mice, but remained unchanged in ApoE^-/-^ mice under both dietary condition (Figure 6B). These findings indicate that ApoE deficiency, particularly under WD conditions, compromises vaccine-induced antibody responses. To further characterize memory T cell responses, we analyzed CD4⁺ and CD8⁺ effector memory T (Tem) cell populations in splenocytes (Figure 6C). Under RCD, vaccination mildly increased Tem frequencies in both genotypes. In WD-fed mice, basal Tem levels were elevated, especially in ApoE^-/-^ mice.

However, CD4⁺ Tem populations did not significantly change post-vaccination, although ApoE^-/-^ mice retained higher baseline levels than ApoE^+/+^ mice (Figure 6C). We next examined antigen-presenting cells to determine whether innate immune components were differentially modulated. Dendritic cell (DC) numbers were not significantly affected by vaccination or ApoE status under RCD (Figure 6D). However, mRNA vaccination elevated CD80 and MHC II expression on DC in ApoE^+/+^ mice, whereas in ApoE^-/-^ mice, only CD80 was upregulated, with a stronger effect under WD. Elevated basal levels of CD80 in ApoE^-/-^ mice may contribute to this limited vaccine-induced activation. Macrophage (MΦ) populations were unaffected by diet or genotype (Figure 6E). Nonetheless, MΦ activation markers (CD80 and MHC II) were significantly upregulated in both genotypes following vaccination, with higher basal expression observed in ApoE^-/-^ mice under both diet conditions and ApoE^+/+^ mice under WD conditions. Taken together, these findings suggested that while ApoE deficiency does not impair vaccine-induced cellular immunity, it selectively suppresses antibody response, particularly under WD conditions. This phenotype may be linked to the elevated basal inflammatory tone observed in ApoE^-/-^ mice, which may skew immune dynamics toward T cell activation while dampening B cell-mediated responses. These results suggest the potential for cholesterol metabolism disorders to alter the balance of vaccine-induced immunity.

### ApoE^-/-^ mice under a Western diet show distinct transcriptional profiles following mRNA vaccination, independent of systemic inflammation

**Figure 7:**
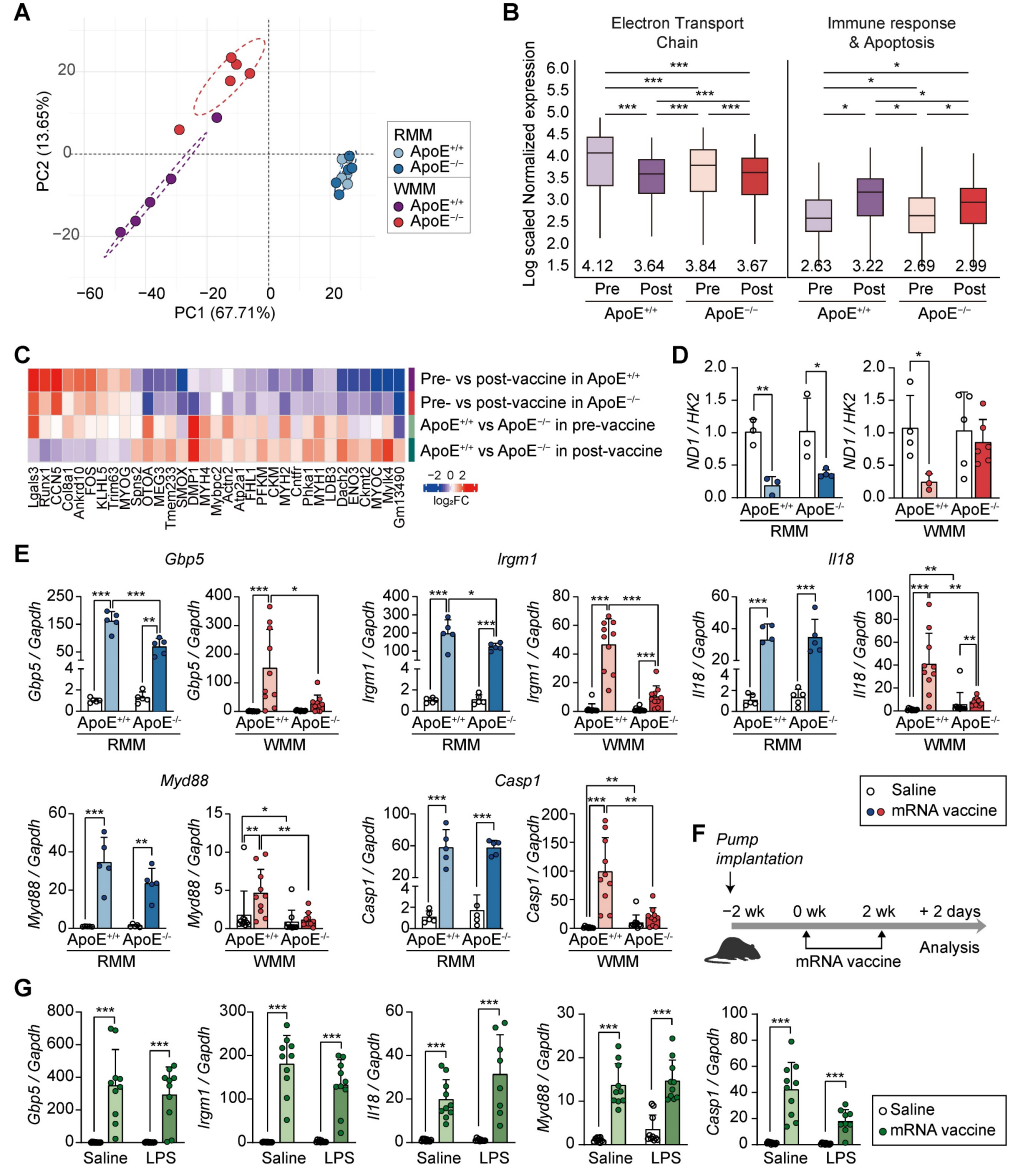
** ApoE-/- mice on a Western diet exhibit attenuated mitochondrial and immune transcriptional responses to mRNA vaccination, independent of systemic inflammation.** (A-E) ApoE+/+ and ApoE-/- mice were fed a regular chow diet (RCD) or Western diet (WD) for 8 weeks, followed by two intramuscular injections of SARS-CoV-2 mRNA vaccine or saline at a two-week interval. (A) Principal component analysis (PCA) of transcriptomic profiles from ApoE+/+ and ApoE-/- mice fed either a regular chow diet (RCD) or a Western diet (WD) following mRNA vaccination. (B) Box plots showing average expression levels of differentially expressed genes (DEGs) related to electron transport chain (ETC) components (left) and immune response and apoptosis (right). (C) Heatmap showing differentially expressed genes (DEGs) overlapping with denervation-associated signatures from public datasets (GSE183802, GSE121589) and a previously published study 42. (D) Mitochondrial DNA content assessed by the ND1/HK2 ratio via qPCR (n = 3, RMM; n = 3-5, WMM). (E) Expression of Gbp5, Irgm1, Il18, Myd88, and Casp1 in quadriceps by qRT-PCR (n = 4-5, RMM; n = 9-10, WMM). (F) Schematic of the chronic inflammation model: C57BL/6 mice were implanted with subcutaneous osmotic pumps delivering either Tween-saline or LPS (300 μg/kg/day) for 4 weeks to induce systemic inflammation. (G) Relative expression level of Gbp5, Irgm1, Il18, Myd88, and Casp1 following mRNA vaccination in LPS-treated and control mice (n = 10, Saline; n = 8-10, LPS). Data are shown as mean (standard deviation). Statistical significance was assessed using a two-tailed Student's t-test; *p < 0.05, **p < 0.01, and ***p < 0.001.

Having established that ApoE deficiency combined with a WD attenuates mRNA vaccine-induced injection site injury (Figure 5) and modulates immune responses (Figure 6), we next performed comprehensive transcriptomic profiling to explore the underlying molecular mechanisms. We identified 1,262 DEGs between ApoE^+/+^ and ApoE^-/-^ mice under both RCD and WD conditions. PCA of these DEGs revealed clear separation between genotypes under WD, but not under RCD, indicating that transcriptomic differences were diet-dependent (Figure 7A). In WD-fed ApoE^-/-^ mice, downregulated genes were enriched for genes involved in the mitochondrial electron transport chain (ETC), while upregulated DEGs were primarily associated with immune responses and apoptosis, following a similar pattern observed in ApoE^+/+^ mice (Figure 7B). However, the magnitude of these transcriptional changes differed substantially between genotypes. To further characterize these gene expression changes, we grouped DEGs (*p* < 0.01) into representative signaling pathways, including chemokine, tumor necrosis factor-α (TNF-α), NF-κB, Caspase, and the mitochondrial OXPHOS-complexes (Figure S7). These pathways were selected based on our prior transcriptomic analyses from macaque and murine models. Vaccination induced upregulation of immune-related pathways such as chemokine, TNF-α, NF-κB, and Caspase signaling in both genotypes, but the fold change was consistently lower in ApoE^-/-^ mice. Similarly, expression of OXPHOS-complexes-related genes decreased significantly in vaccinated WD-fed ApoE^+/+^ mice, while ApoE^-/-^ mice showed only modest downregulation (Figure S7). Notably, transcriptional patterns in ApoE^+/+^ mice closely resembled those reported in recent single-cell transcriptomic datasets from vaccine injection sites (Table S1 and S2) 40. While both genotypes showed similar directional changes following vaccination, the magnitude of induction or repression was reduced in ApoE^-/-^ mice. Specifically, mRNA vaccination caused a substantial reduction in ETC-related genes in ApoE^+/+^ mice under WD (*p* < 0.0001), reaching expression levels comparable to the pre-existing suppressed baseline observed in ApoE^-/-^ mice. Conversely, ApoE^-/-^ mice demonstrated only moderate decreases in OXPHOS-complexes gene expression after vaccination (*p* < 0.001), consistent with their already attenuated baseline. Immune response and apoptosis-related gene expression were also upregulated in both genotypes following vaccination, but the increase was significantly greater in ApoE^+/+^ mice (*p* < 0.01). In support of the transcriptomic findings, vaccinated ApoE^-/-^ mice under WD conditions were contained fewer TUNEL-positive cells at the injection sites compared to ApoE^+/+^ mice, indicating reduced apoptosis (Figure S8). Together, these results indicate that ApoE^-/-^ mice displayed an attenuated response that nevertheless reached comparable post-vaccination gene expression levels. These findings suggest that ApoE deficiency under WD alters the dynamic range of immune and mitochondrial gene expression in response to mRNA vaccination, potentially explaining the reduced tissue damage observed. In addition, comparative transcriptomic analysis revealed that ApoE^-/-^ mice displayed significantly fewer denervation-associated transcriptional changes following vaccination than ApoE^+/+^ mice (Figure 7C). Given the central role of mitochondrial function in the observed transcripttional divergence, we assessed mitochondrial DNA copy number. Unlike ApoE^+/+^ mice, which exhibited significant alterations, ApoE^-/-^ mice under WD showed no change in mitochondrial content post-vaccination (Figure 7D). To identify specific genes responsible for the attenuated transcriptional response in ApoE^-/-^ mice, we identified 12 most significantly differentially expressed genes between the mRNA-vaccinated ApoE^+/+^ and ApoE^-/-^ mice under WD conditions. These included key immune regulators such as *Gbp5, Irgm1, Il18, Myd88*, and *Casp1* (Figure 7E and Figure S9A). Differential expression of these genes was confirmed qRT-PCR (Figure 7E and Figure S9A).

Notably, under RCD conditions, these genes showed no significant expression differences between genotypes. KEGG pathway analysis revealed that the differentially expressed genes were predominantly enriched in the NOD-like receptor signaling pathway, suggesting involvement in innate immune sensing. To determine whether these transcriptional changes were driven by chronic inflammation resulting from cholesterol overload, rather than being a direct consequence of ApoE deficiency, we established a separate mouse model of systemic inflammation using an LPS-releasing pump in combination with mRNA vaccination (Figure 7F). Previous studies have shown that chronic inflammation induced by an LPS-releasing pump leads to reduced antibody production 24, mirroring the humoral immune response observed in ApoE^-/-^ mice. In our system, the LPS-induced model increased *Ccl7* and *Myog*, both markers for muscle inflammation and regeneration (Figure S9B). However, the LPS-induced inflammation model showed a distinct transcriptional pattern compared to ApoE^-/-^ mice: whereas *Gbp5*, *Irgm1*, *Il18*, *Myd88*, and *Casp1* were downregulated in ApoE^-/-^ mice under WD, these genes were markedly increased following LPS treatment (Figure 7G). Together, these findings indicate that ApoE deficiency shapes the transcriptional response to mRNA vaccination under WD conditions, in a manner not replicated by inflammation alone, and may contribute directly to reduced tissue damage at the injection site.

### Differential cardiac responses to mRNA vaccination in ApoE^+/+^ and ApoE^-/-^ mice

**Figure 8:**
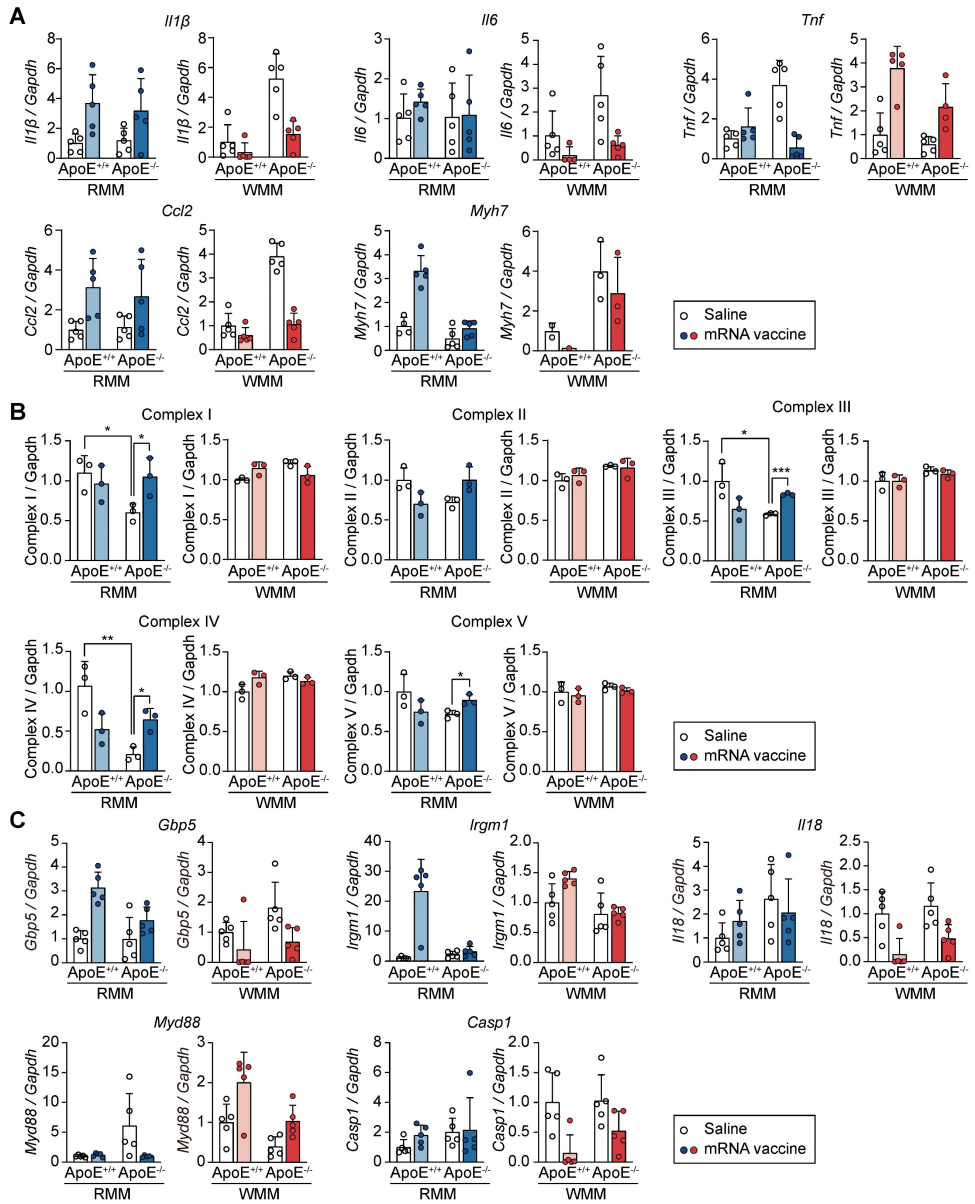
** mRNA vaccination elicits limited transcriptional responses in the heart compared to the injection site.** ApoE+/+ and ApoE-/- mice were fed either a regular chow diet (RCD) or Western diet (WD) for 8 weeks. Two mRNA vaccine doses were administered two weeks apart, and hearts were collected for analysis 48 h after the final dose. (A) Relative expression levels of Il1β, Il6, Tnf, Ccl2, and Myh7 in the heart tissue measured by qRT-PCR and normalized to Gapdh (n = 4-5, RMM; n = 1-5, WMM). (B) Quantitative analysis of mitochondrial oxidative phosphorylation (OXPHOS) complexes I-V in heart lysates. Band intensities were measured using ImageJ (n = 3, RMM; n = 3, WMM). (C) Relative expression levels of Gbp5, Irgm1, Il18, Myd88, and Casp1 in heart assessed by qRT-PCR (n = 5, RMM; n = 5, WMM). Data are presented as mean (standard deviation). Two-tailed Student's t-test was used to determine significance; *p < 0.05, **p < 0.01, and ***p < 0.001.

To evaluate whether the immunological and transcriptional effects of mRNA vaccination extended beyond the injection site, we analyzed cardiac tissue for changes in inflammation, injury, and mitochondrial function. We specifically assessed the expression of inflammatory cytokines (*Il1β*, interleukin-6 (*Il6*), *Tnf,* C-C motif chemokine ligand 2 (*Ccl2*)) and the cardiac injury marker *Myh7*, in ApoE^+/+^ and ApoE^-/-^ mice following mRNA vaccination (Figure 8A). In ApoE^+/+^ mice on RCD, vaccination induced mild, non-significant increases in* Il1β, Il6, Ccl2* and *Myh7*, contrasting with the robust immune and injury-related transcriptional responses observed at the injection site. Under WD conditions, expression of these genes was further reduced following vaccination in ApoE^+/+^ mice, suggesting diet-dependent modulation of cardiac immune activation. *Tnf* displayed a unique genotype- and diet-specific expression pattern: it was upregulated in WD-fed ApoE^+/+^ mice but downregulated in RCD-fed ApoE^-/-^ mice. This differential response illustrates the complex interplay between genetic background and dietary context in shaping cardiac immune response post-vaccination. To evaluate potential changes in mitochondrial structure and abundance, we measured the protein levels of mitochondrial oxidative phosphorylation complexes I-V. No significant changes were observed regardless of genotype or diet, suggesting preserved mitochondrial integrity in the heart following mRNA vaccination (Figure 8B and Figure S10). To determine whether genes involved in the attenuated response at the injection site (Figure 7E) were similarly regulated in cardiac tissue, we examined the expression of *Gbp5*, *Irgm1*, *Il18*, *Myd88*, and *Casp1*. Overall, the cardiac transcriptional changes were more limited and variable. In RCD-fed ApoE^+/+^ mice, modest increases in *Gbp5* and *Irgm1* were observed post-vaccination, while *Il18* and *Casp1* remained unchanged. *Myd88* was elevated at baseline in ApoE^-/-^ mice with no further increase upon vaccination. Under WD conditions, transcriptional responses were modest across genotypes, with no clear ApoE-dependent differences (Figure 8C). Together, these results demonstrated that cardiac tissue exhibits only modest and largely non-significant changes in response to mRNA vaccination, in contrast to the robust transcriptional activation observed at the injection site.

## Discussion

mRNA vaccines represent a powerful tool in public health, offering rapid and flexible protection against emerging infectious threats. While their efficacy and safety have been widely validated, questions remain about their localized effects on non-target tissues—particularly at the injection site, which serves as the primary site of vaccine delivery and initial immune activation. In this study, we demonstrated that intramuscular mRNA vaccination induces marked tissue damage at the injection site, including mitochondrial dysfunction, inflammatory activation, and increased apoptosis. These findings prompt further investigation into the mechanisms that underlie tissue-specific responses to mRNA vaccines. Although adverse injection site-related events are relatively rare, emerging clinical reports of post-vaccination myalgia and biopsy-confirmed myositis suggest that such responses may be underrecognized. A recent review identified 49 cases of post-vaccination myositis predominantly linked to mRNA platforms 7, supporting the relevance of investigating vaccine-associated injection site effects. Our findings provide mechanistic insight into how genetic and metabolic status, particularly ApoE deficiency and dietary conditions, modulate injection site-specific responses to mRNA vaccination.

Transcriptomic analyses revealed key pathways involved in immune responses, apoptosis, and mitochondrial dysfunction. Notably, gene expression profiles in NOD-like receptor signaling and mitochondrial ETC in ApoE^+/+^ mice closely resembled findings from recent single-cell transcriptomic datasets 40. Despite the differences in sampling time points and analytical methods (bulk mRNA sequencing vs. single-cell mRNA sequencing), this concordance reinforces the reproducibility of molecular signatures and supports the relevance of our findings in understanding injection site-specific vaccine responses.

Unlike conventional vaccines, mRNA vaccines appear to trigger robust innate immune response at the injection site, which may underlie the tissue alterations observed in this study. This contrasts with adenovirus-based vector vaccines, which tend to elicit different local inflammatory profiles 40, 48, 49. These local effects are likely shaped by both antigen expression and LNP-mediated delivery. Prior studies have shown that LNPs can influence cellular stress pathways through membrane perturbation, calcium imbalance, and mitochondrial dysfunction, depending on the cellular and metabolic context 50-54.

Our analysis further revealed a significant overlap between the phenotypes observed in mRNA-vaccinated muscle at the injection site and those associated with denervation, including similarities in gene expression, morphological abnormalities, and mitochondrial dysfunction 55, 56. Transmission electron microscopy confirmed these findings, revealing swollen intermyofibrillar (IMF) mitochondria with disrupted cristae, while subsarcolemmal (SS) mitochondria remained largely intact. These ultrastructural abnormalities were accompanied by reduced succinate dehydrogenase (SDH; complex II) staining and decreased mitochondrial DNA copy number, indicating mitochondrial enzymatic dysfunction and loss of mitochondrial content. Alongside these alterations, reduced expression of sarcomeric and cytoskeletal proteins likely contributed to impaired contractile function and decreased grip strength.

To clarify whether the protective phenotype observed in ApoE^-/-^ mice was due to chronic inflammation associated with lipid accumulation or an intrinsic effect of ApoE deficiency, we employed an LPS-induced inflammation model. While LPS treatment is associated with systemic immune activation, our model did not recapitulate the transcriptional signature observed in ApoE-deficient mice. Markers such as *Gbp5*, *Irgm1*, *Il18*, *Myd88*, and *Casp1* remained unaffected, strongly suggesting that ApoE itself—rather than inflammation itself—is a key determinant of injection site response.

Our findings suggest that ApoE deficiency under a WD reduces inflammation and tissue damage at the injection site following mRNA vaccination, independent of inflammation directly attribute to lipid accumulation. This protective effect may originate from intrinsic adaptations in muscle cells at the injection site. In WD-fed ApoE-deficient mice, chronic exposure to lipid excess and metabolic stress appears to induce compensatory adaptations in muscle fibers, including enhanced fatty acid oxidation, preserved OXPHOS capacity, and increased expression of mitochondrial maintenance genes 57-60. These adaptations are accompanied by attenuation of vaccine-induced downregulation of mitochondrial ETC genes (Figure 7B), along with reduced mitochondrial DNA loss (Figure 7D) and lower apoptosis levels (Figure S8), suggesting that metabolic stress may precondition the injection site to better tolerate acute mitochondrial perturbations. In contrast, under RCD conditions, muscle fibers in ApoE-deficient mice retain a glycolytic-dominant metabolic profile without prior mitochondrial adaptation 57, 59-61, rendering them more susceptible to OXPHOS gene downregulation, cristae disruption, and immune-mediated injury upon vaccination. Thus, ApoE deficiency alone does not confer protection at the injection site unless accompanied by metabolic preconditioning that promotes mitochondrial resilience and prevents structural deterioration.

Despite significant changes at the injection site, the cardiac response to mRNA vaccination was minimal. Although previous studies have reported cardiac adverse events following mRNA vaccination in inflammatory settings 24, 62, we observed no such effects in our model. Inflammatory gene expression in the heart remained modest, particularly under WD conditions. This tissue specificity indicates that transcriptional responses to mRNA vaccination may differ across organs, potentially reflecting local regulatory mechanisms. These findings suggest that genetic and metabolic context modulate localized responses to mRNA vaccination, with ApoE status playing a key role at the injection site. Long-term studies are needed to evaluate whether repeated dosing or changes in delivery platforms exacerbate mitochondrial damage or impair regenerative capacity. Future interventions aimed at restoring mitochondrial homeostasis—such as enhancing biogenesis or stabilizing membrane potential—may help mitigate tissue injury while preserving vaccine immunogenicity.

Given that the protective phenotype associated with ApoE deficiency emerged only under WD conditions, our findings raise the possibility that transient inhibition of ApoE activity may reduce vaccine-induced tissue injury in metabolically stressed individuals. Such a strategy could preserve mitochondrial integrity and attenuate inflammatory signaling at the injection site without impairing systemic immune responses. Therapeutic modulation of ApoE during vaccination may therefore represent a viable approach to improve tolerability and safety in populations at risk for lipid-driven mitochondrial vulnerability.

## Conclusion

This study demonstrates that mRNA vaccination induces mitochondrial dysfunction and tissue injury at the injection site, driven by denervation-like transcriptional alterations and local inflammatory responses. ApoE deficiency under metabolic stress conditions provides protection against these effects, revealing a previously unrecognized connection between lipid metabolism and vaccine-induced tissue responses. These results suggest that metabolic and genetic factors may influence individual vulnerability to injection site injury. Moreover, metabolic preconditioning could serve as a strategy to improve vaccine tolerability, supporting the development of personalized vaccination approaches that maintain immune efficacy while reducing local adverse effects, particularly in individuals with underlying metabolic disorders.

## Supplementary Material

Supplementary methods, figures and tables.
