# Supplementary material for: ApoE deficiency protects from mRNA vaccine-induced mitochondrial dysfunction at the injection site under metabolic stress
Source: Theranostics. 2025 Aug 16;15(17):8964–84. doi: 10.7150/thno.119545 (PMC12439255; doi:10.7150/thno.119545)
Supplement: Supplementary file 1 — Supplementary methods, figures and tables. [file thnov15p8964s1.pdf]

Supplemental Experimental Section

Quantitative real-time polymerase chain reaction (qRT-PCR)

For RNA isolation, quadriceps tissues and hearts were lysed in Lysis Reagent (QIAGEN, Hilden, Germany) and homogenized using TissueLyser II (QIAGEN). Total RNA was extracted using the RNeasy Mini Kit or Midi kit (QIAGEN), following the manufacturer's protocol. Complementary DNA (cDNA) was synthesized using a TOPscriptTM RT DryMIX kit (Enzynomics, Daejeon, Republic of Korea). qRT-PCR was carried out using the TOPrealTM qPCR 2× PreMIX (SYBR Green with high ROX) kit (Enzynomics) on a CFX384 C1000 Thermal Cycler (Bio-Rad). Detailed primer sequences are provided in Table S2. mRNA expression levels were quantified and presented as fold changes relative to the saline-injected control group.

Protein extraction and Western blot analysis

Proteins were extracted from frozen heart samples by homogenizing the tissues in ice-cold RIPA buffer (50 mM Tris, pH 8.0, 0.1% SDS, 1% NP-40, 150 mM NaCl, 0.5% Sodium-deoxycholate). The homogenates were incubated on ice for 30 min and then centrifuged at 12,000 × g for 15 min at 4 °C to collect the supernatants containing total protein extracts. Approximately 20 µg of protein from each sample was resolved using 10% SDS-polyacrylamide gel electrophoresis and transferred to polyvinylidene fluoride (PVDF) membranes. The membranes were incubated overnight at 4 °C with primary antibodies specific to OXPHOS complexes (Thermo Fisher Scientific, Waltham, CA, USA) and glyceraldehyde 3-phosphate dehydrogenase (GAPDH) (Cell Signaling Technology, Danvers, MA, USA). Following primary antibody incubation, the membranes were incubated for 1 h at room temperature with horseradish peroxidase (HRP)-conjugated secondary antibodies. Protein bands were visualized using enhanced chemiluminescence (ECL) substrate (Thermo Fisher Scientific, CA, USA). Images were captured

using the ChemiDoc XRS+ system (Bio-Rad, Hercules, CA, USA), and band intensities were quantified using ImageJ software.

Creatine kinase activity assay

Serum was processed using a Creatine Kinase Activity Assay Kit (Hoffmann-La Roche, Basel, Switzerland) following the manufacturer's protocol. Creatine kinase activity was measured by assessing absorbance at wavelengths of 546/340 nm using the Cobas ® c502 module multimode Microplate Reader (Roche Diagnostics, Mannheim, Germany). All procedures were performed in accordance with the standard instructions provided by the manufacturer.

Troponin I assay

Troponin I (TnI) levels were measured using a chemiluminescent immunoassay (CLIA) kit (Cat. No. 15243092, Siemens, Berlin, Germany) on the Atellica® IM Analyzer platform following the manufacturer′s instructions. To quantify serum TnI levels, values were expressed as fold change relative to the saline-injected control group.

Measurement of triglyceride and total cholesterol

Blood samples were centrifuged to separate the plasma, and experiments were conducted according to Triglyceride and Total cholesterol protocols. L-type Triglyceride M and LabAssay™ cholesterol kits (FUJIFILM Wako Shibayagi Co., Ltd., Gunma Prefecture, Japan) were used for measurements at a wavelength of 600 nm using a Microplate Reader (Molecular Devices, California, USA).

Bioluminescence imaging

C57BL/6J mice were used to assess the mRNA expression levels. The mRNA vaccine was injected into the quadriceps muscle at a concentration of 5 μg/40 μl. After 3 h, 150 mg/kg l-luciferin (Promega, Madison, WI, USA) was intraperitoneally injected. Measurements were conducted

using an in vivo optical imaging system (Spectral Instruments Imaging), and the luminescence signal of the region of interest (ROI) was analyzed using the Aura64 software.

Apoptosis detection using TUNEL staining

Apoptosis was assessed using the DeadEnd™ Fluorometric TUNEL System (G3250; Promega, Madison, WI, USA) according to the manufacturer's protocol. After staining, slides were mounted with DAPI-containing mounting medium (Vector Laboratories, Newark, CA, USA) and analyzed using a Leica Thunder imager (Leica Microsystems, Wetzlar, Germany).

Analysis of denervation-related genes and comparative expression patterns using scRNA-seq dataset GSE239674

To investigate the expression patterns of denervation-related genes, 66 marker genes were collected from three studies, each reporting significant expression changes during denervation in *Mus musculus* muscle tissue [1-3]. These genes were selected based on the fold change and p-value criteria reported in the respective studies or explicitly annotated as DEGs. Specifically, an absolute log2FoldChange ≥ 1 and $p < 0.01$ were applied in the studies by Castets, P. *et al* [1] and Henze, H. *et al* [3]. For the dataset from Alsaigh, T. *et al* [2], the Wilcoxon Rank Sum test was used with parameters min.pct = 0.25 and logfc.threshold = 0.25. Using these 66 genes, denervation-related DEGs were identified across pre- and post-vaccination conditions in WMM, RMM, and RMF groups. To compare the expression patterns of NOD-like receptor signaling and mitochondrial ETC genes in this study with those from a previous study, we analyzed the publicly available dataset (GSE239674) [4] from the Gene Expression Omnibus (GEO). This dataset, derived from vaccinated *Mus musculus*, used single-cell RNA sequencing (scRNA-seq) to profile muscle tissues, lymph nodes, and peripheral blood cells. The scRNA-seq data were processed using Seurat v5.1.0 [5] to generate a count table, with gene expression values averaged across all

cells within each sample. DEGs were identified following the same protocol used for RNA-seq data analysis in this study.

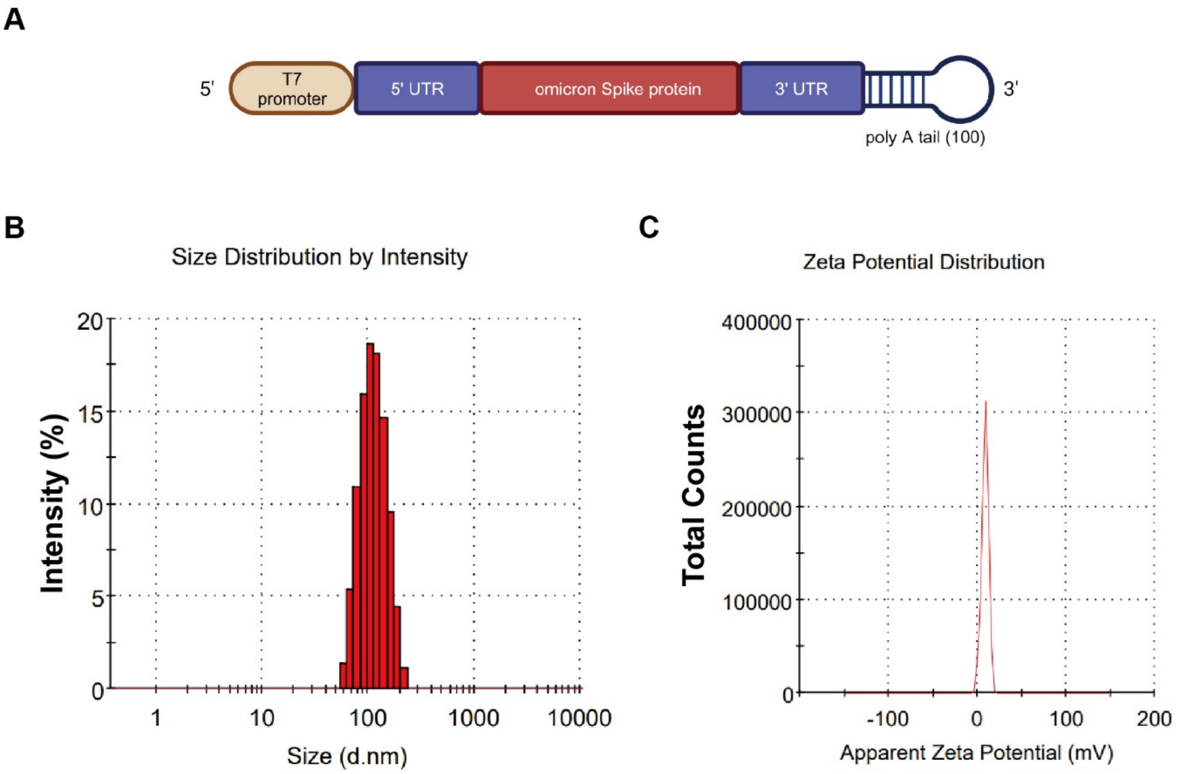

Sample	Z-Ave (d.nm)	PDI	Zeta Potential (mV)
LNP128-S-omicron	108.2±1.250	0.078±0.006	9.32±1.02

Figure S1. Physicochemical characterization of mRNA vaccine

(A) Schematic representation of the mRNA vaccine used in the experiments. (B-C) Size and zeta potential of the lipid nanoparticles comprising the mRNA. The data are presented as histograms representing the mean particle size from at least three independent measurements.

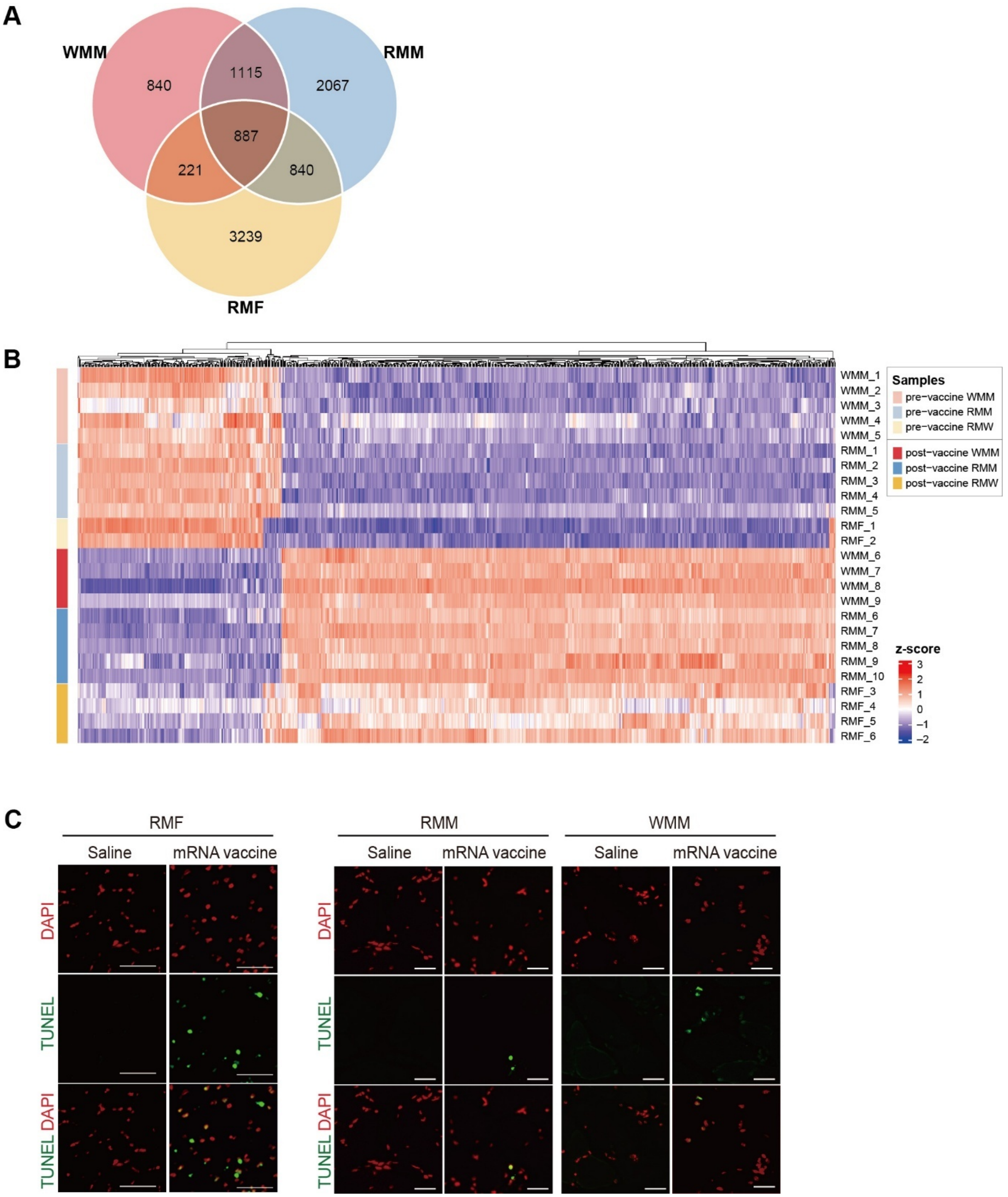

Figure S2. Shared transcriptomic responses and apoptotic signatures in muscle following mRNA vaccination across species and dietary models

(A) Venn diagram of differentially expressed genes (DEGs) identified in *Mus musculus* fed a regular chow diet (RMM), *Mus musculus* fed a Western diet (WMM), and *Macaca fascicularis* fed a regular chow diet (RMF). A total of 887 DEGs were common across all three groups. (B) Heatmap visualizing the expression patterns of the 887 shared DEGs in RMM, WMM, and RMF groups. (C) Detection of apoptotic cells in quadriceps muscle following mRNA vaccination in *Macaca fascicularis* and *Mus musculus* using TUNEL staining. DAPI staining, which normally appears blue, is shown in red using pseudo-coloring to enhance contrast in the merged images. Scale bar: 50 μm.

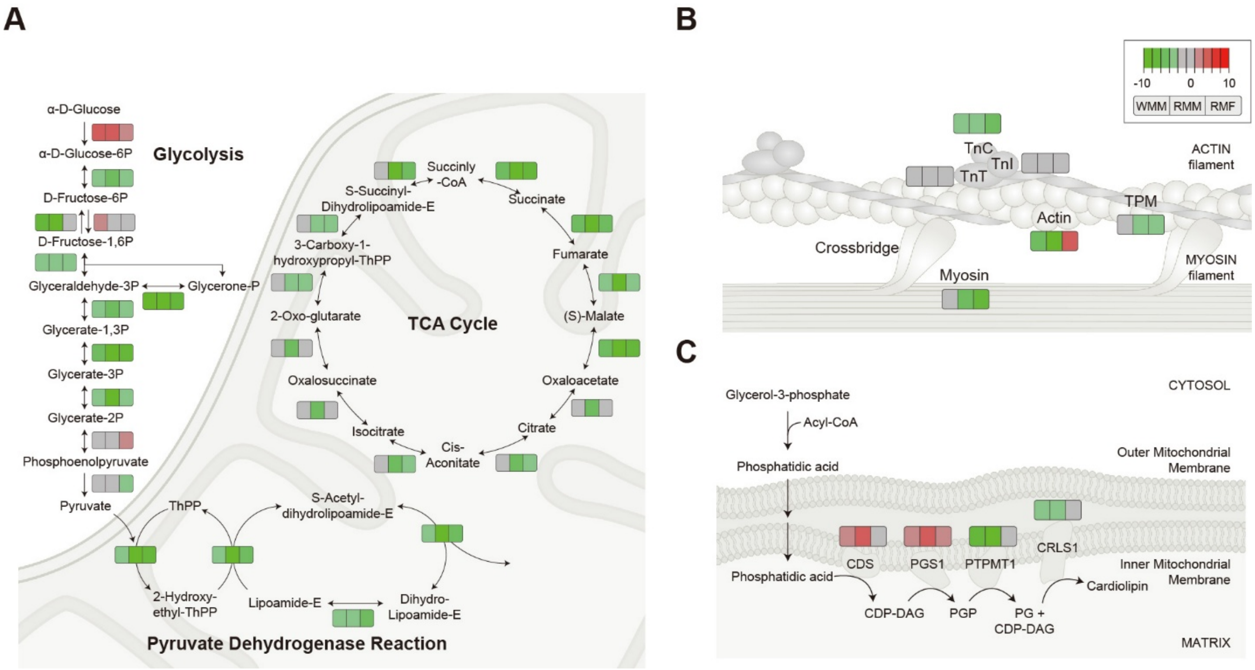

Figure S3. mRNA vaccination alters genes involved in metabolism, muscle structure, and mitochondrial lipid biosynthesis

(A) Visualization of pathway-level changes in metabolic processes, including glycolysis, the pyruvate dehydrogenase reaction, and the tricarboxylic acid (TCA) cycle. (B) Pathway analysis showing downregulation of genes related to muscle structure, particularly those associated with the cardiomyopathy pathway. These include troponin C (TnC), troponin I (TnI, cardiac isoform *Tnni3*), tropomyosin (TPM), troponin T (TnT), skeletal muscle actin (*ACTA1*), and members of the myosin heavy chain (*MYH*) family. (C) Transcriptional changes in genes involved in cardiolipin biosynthesis following mRNA vaccination. Affected genes include CDP-diacylglycerol synthase (*CDS1*), phosphatidylglycerophosphate synthase 1 (*PGS1*), protein tyrosine phosphatase, mitochondrial 1 (*PTPMT1*), and cardiolipin synthase 1 (*CRLS1*).

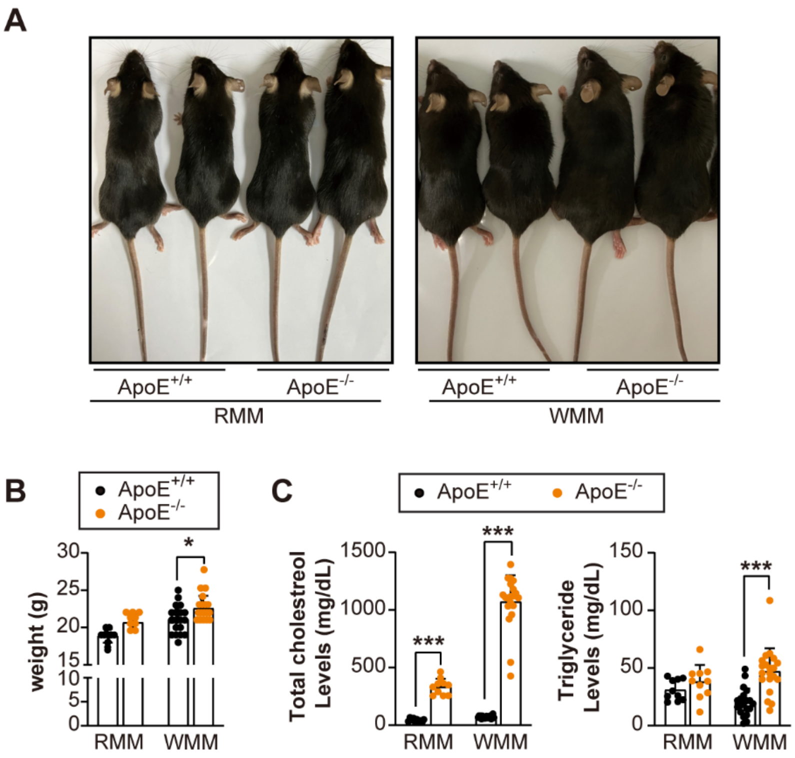

Figure S4. Characterization of hypercholesterolemic ApoE$^{-/-}$ and ApoE$^{+/+}$ mouse models under dietary challenge

(A) Representative images of ApoE$^{+/+}$ and ApoE$^{-/-}$ mice fed a regular chow diet or Western diet for 8 weeks, followed by two mRNA vaccine doses administered 2 weeks apart. (B) Body weights corresponding to dietary groups shown in (A). (n = 10-18, ApoE$^{+/+}$; n = 9-18, ApoE$^{-/-}$). (C) Plasma cholesterol and triglyceride levels measured from blood samples collected from the same experimental groups (n = 10-19, ApoE$^{+/+}$; n = 9-18, ApoE$^{-/-}$). Data are represented as mean (standard deviation). Statistical significance was assessed by a two-tailed Student's t-test; *$p < 0.05$, **$p < 0.01$, and ***$p < 0.001$.

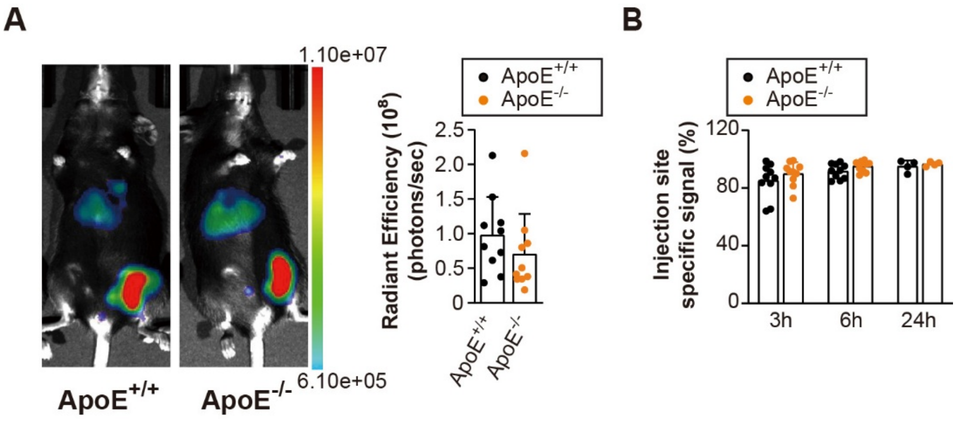

Figure S5. mRNA biodistribution of ApoE$^{-/-}$ and ApoE$^{+/+}$ mouse models.

(A) Luminescence at the injection site was measured 3 h after D-luciferin injection using Aura64 software in ApoE$^{+/+}$ and ApoE$^{-/-}$ mice (n = 10 per group). (B) Time-course analysis of luminescence intensity at the indicated time points. Injection site-specific signal was quantified as the ratio of local to total body luminescence (n = 10, ApoE$^{+/+}$; n = 4-10, ApoE$^{-/-}$). Data are presented as mean (standard deviation). Statistical significance was determined using a two-tailed Student's t-test; *$p < 0.05$, **$p < 0.01$, and ***$p < 0.001$.

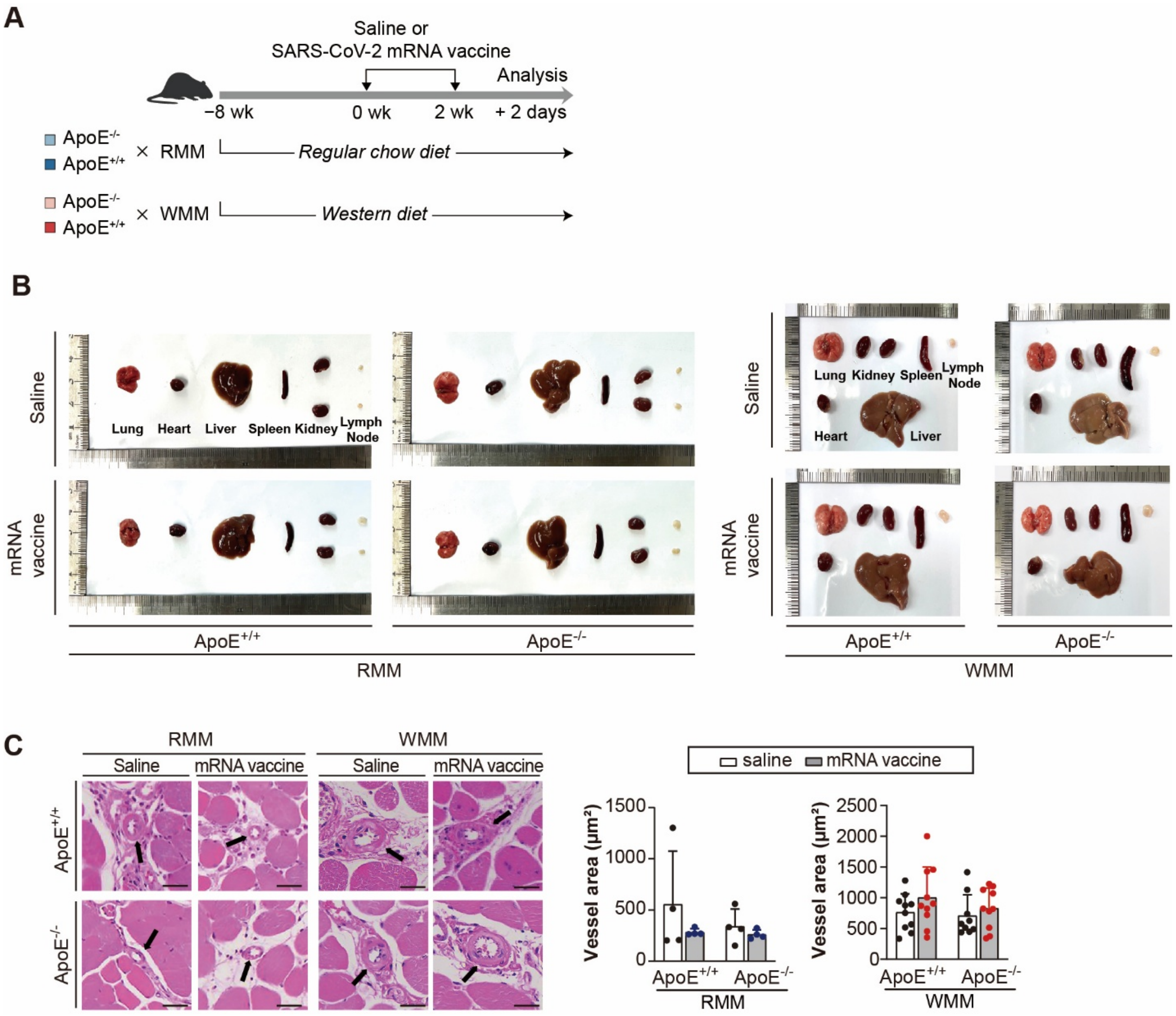

Figure S6. Experimental design and quadriceps vascular area analysis in ApoE$^{+/+}$ and ApoE$^{-/-}$ mice after mRNA vaccination

(A) Experimental design for ApoE$^{+/+}$ and ApoE$^{-/-}$ mice. Mice were fed either a regular chow diet (RCD) or a Western diet (WD) for 8 weeks and received two mRNA vaccine doses at 2-week intervals. Quadriceps were collected 48 h after the final injection. Schematic of the experimental timeline for ApoE$^{+/+}$ and ApoE$^{-/-}$ mice used in Figure 5, Figure 6, Figure 7A-7E, and Figure 8. (B) Tissues (lung, liver, heart, spleen, kidney, and lymph nodes) were isolated 48 h after the final vaccine injection for downstream analysis. (C) Vessel area in quadriceps after mRNA vaccination

was measured using ImageJ. (n = 4, RMM; n = 9-10, WMM). Scale bars: 40 μm. Data are presented as mean (standard deviation). Statistical significance was assessed by a two-tailed Student's t-test; *$p < 0.05$, **$p < 0.01$, and ***$p < 0.001$.

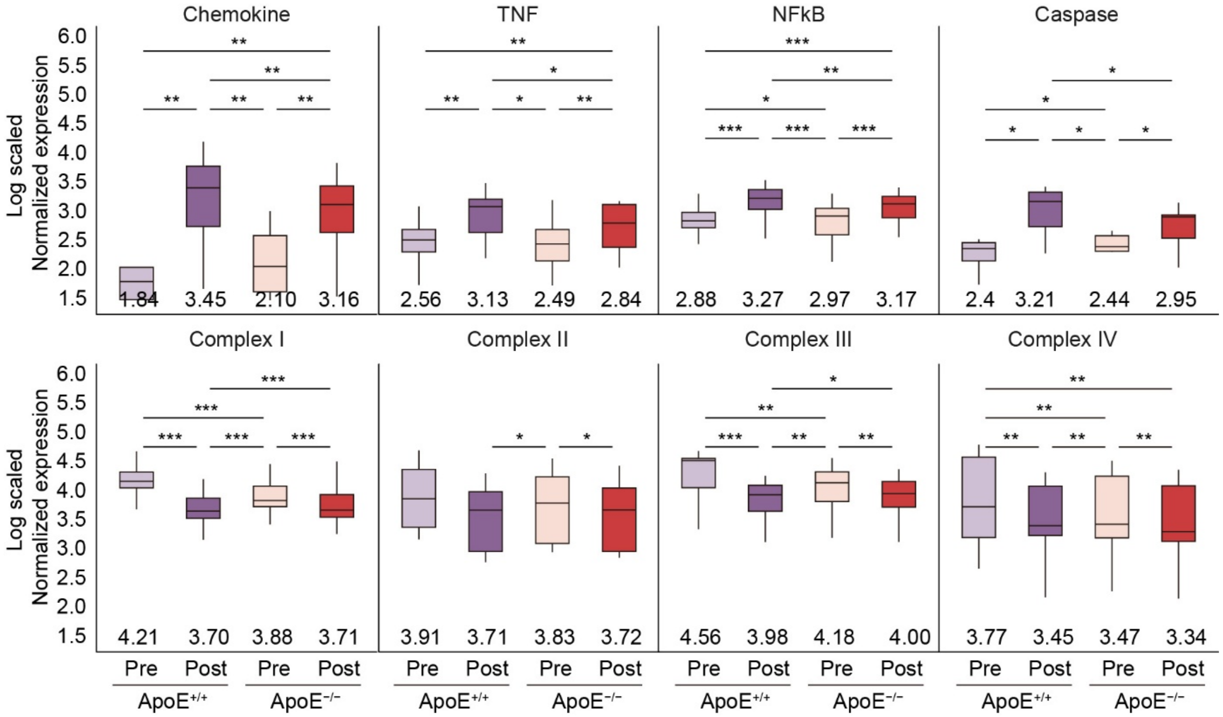

Figure S7. ApoE deficiency mice under Western diet exhibit attenuated transcriptional responses to mRNA vaccination

Transcriptomic profiles from ApoE$^{-/-}$ mice fed a Western diet (WD) following mRNA vaccination. Box plots illustrating expression changes of differentially expression genes (DEGs) involved in chemokine signaling, TNF, NF-κB, Caspase pathways, and mitochondrial oxidative phosphorylation complexes. Median values are indicated in each plot. Statistical significance was assessed by a two-tailed unpaired Student's t-test; *$p < 0.05$, **$p < 0.01$, and ***$p < 0.001$.

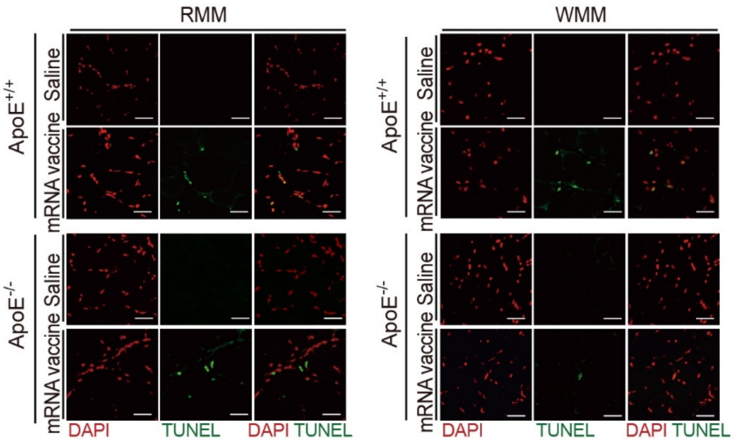

Figure S8. TUNEL-based detection of apoptosis in ApoE$^{+/+}$ and ApoE$^{-/-}$ mice following mRNA vaccination

ApoE$^{+/+}$ and ApoE$^{-/-}$ mice were fed either a regular chow diet (RCD) or a Western diet (WD) for 8 weeks, followed by two intramuscular doses of SARS-CoV-2 mRNA vaccine administered 2 weeks apart. Tissues were collected 48 h after the final dose. Apoptotic cells in quadriceps were detected by TUNEL staining. For improved visualization, DAPI was pseudo-colored in red rather than its native blue fluorescence. Scale bars: 50 µm.

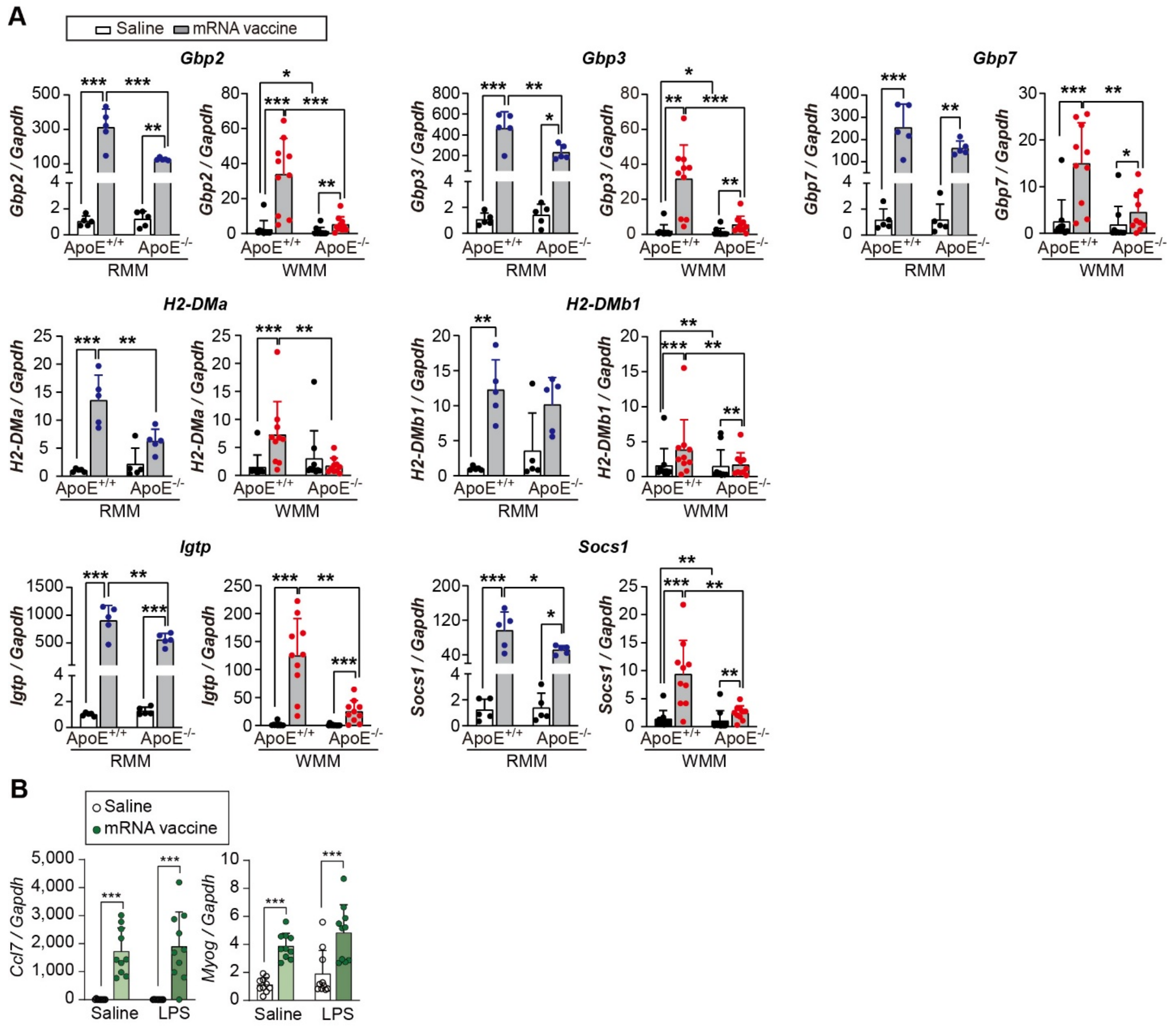

Figure S9. ApoE deficiency and Western diet alter local mRNA vaccine responses, distinct from systemic inflammation

ApoE$^{+/+}$ and ApoE$^{-/-}$ mice were fed either a regular chow diet (RCD) or a Western diet (WD) for 8 weeks, followed by two intramuscular doses of SARS-CoV-2 mRNA vaccine at 2-week intervals. Quadriceps muscles were harvested 48 h after the final injection. (A) Total RNA was extracted from muscle tissue, and expression levels of *Gbp2, Gbp3, Gbp7, H2-DMa, H2-DMb1, Igtp,* and *Socs1* were quantified by qRT-PCR and normalized to *Gapdh.* These genes were selected based

on the largest expression differences observed between $ApoE^{+/+}$ and $ApoE^{-/-}$ mice under Western diet conditions (n = 5, RMM; n = 9-10, WMM). Mice were implanted with subcutaneous osmotic pumps delivering either Tween-saline or LPS for 4 weeks to induce systemic inflammation. (B) Expression of *Ccl7* and *Myog* in quadriceps of LPS-treated and control mice by qRT-PCR and normalized to *Gapdh* (n = 10, Saline; n = 10, LPS). Data are presented as mean (standard deviation). Statistical significance was assessed by a two-tailed Student's t-test; $*p < 0.05$, $**p < 0.01$, and $***p < 0.001$.

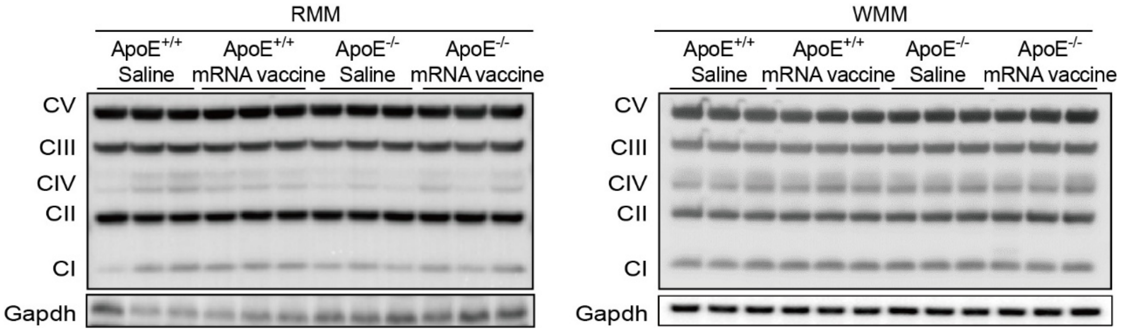

Figure S10. mRNA vaccination does not alter cardiac oxidative phosphorylation (OXPHOS) complex I–V protein levels in ApoE$^{+/+}$ and ApoE$^{-/-}$ mice under regular or Western diet

ApoE$^{+/+}$ and ApoE$^{-/-}$ mice were fed either a regular chow diet (RCD) or a Western diet (WD) for 8 weeks, followed by two intramuscular doses of SARS-CoV-2 mRNA vaccine at 2-week intervals. Hearts were collected 48 h after the second dose. Cardiac tissue lysates were analyzed by western blot to assess protein levels of OXPHOS complexes I–V: Complex I (CI), NADH: ubiquinone oxidoreductase; Complex II (CII), Succinate dehydrogenase; Complex III (CIII), Cytochrome bc_1 complex; Complex IV (CIV), Cytochrome c oxidase; Complex V (CV), ATP synthase (n = 3, RMM; n = 3, WMM).

Table S1. Fold changes of differentially expressed genes (DEGs) in the NOD-like receptor signaling pathway overlapping with public dataset GSE239574

This table presents the fold changes of DEGs within NOD-like receptor signaling pathway identified in mRNA-vaccinated $ApoE^{+/+}$ mice, that overlap with those in the publicly available dataset GSE239574. The overlapping DEGs are primarily associated with NOD-like receptor signaling, indicating conserved molecular responses to mRNA vaccination across datasets.

Gene_Symbol	This_study	GSE239574
Ccl12	9.3562648	1.28499186
Ccl2	8.6887075	0.06401388
Irgm2	6.311012	2.41961552
Gbp2	6.7064794	2.5119833
Irgm1	6.2119636	1.85906421
Gbp5	6.456915	2.56731332
Gbp3	6.3957519	2.28687364
Gbp7	5.9961026	2.58547876
Il6	6.0931204	-0.78714953
Irf7	6.2620702	3.91682986
Oas1g	5.6826037	4.69581313
Mefv	5.7527061	0.55054525
Oas1a	5.4163763	3.79514883
Cxcl1	4.6273745	-1.31667961
Ifi204	4.3881436	1.41131026
Casp4	4.6010153	0.71596479
Nlrp12	2.4891413	0.14533821
Oas1b	3.9281567	NA
Trpv2	3.328426	0.08226662
Pstpip1	4.1522069	1.1077262
Sting1	3.7992222	NA
Il18	3.5485536	0.73759693
Trpm2	3.5277239	-0.1505358
Ikbke	3.2455656	1.08349683
Oas2	3.0805863	4.6222176
Cyba	3.1222641	-0.03491028
Aim2	2.7007952	0.86659656
Prkcd	2.6581458	0.30121557
Naip2	2.8458629	0.33056201
Card9	3.0562563	-0.91584337
Gpsm3	2.6652005	0.36472613

Nod2	2.9731267	0.48459128
Gsdmd	2.7319058	0.47625754
Nlrp1a	2.7619122	2.36843819
Nod1	2.3257686	0.69552243
Plcb2	1.9647559	0.54189659
Ripk2	1.6921547	0.15663741
Naip6	1.6789701	0.24854571
Tbk1	1.1905098	0.95339751
Rnasel	1.7982216	1.01238718
Naip5	1.7626347	-0.19566451
Plcb1	1.5111547	-1.15268366
Nlrc4	1.7012553	0.10138895
Nlrp1b	1.5744795	1.11477888
Casr	-0.9449046	NA
Mfn1	-1.1523945	-0.42498493
Map1lc3a	-1.3249195	-1.59961116

Table S2. Differentially expressed genes (DEGs) in the mitochondrial electron transport chain overlapping with public dataset GSE239574

This table summarizes the fold changes of DEGs related to mitochondrial electron transport identified in mRNA-vaccinated ApoE+/+ mice. These genes overlap with those found in the public dataset GSE239574 and reflect conserved changes in mitochondrial bioenergetic pathways following mRNA vaccination.

Gene_Symbol	This_study	GSE239574
Cox15	0.6861213	0.836610451
ND6	-0.9758515	NA
Ndufs1	-0.9202085	-0.072567683
Ttc19	-0.9011791	-0.705234208
Atp5b	-0.9258798	-0.352432096
Atp5a1	-0.9910696	-0.250387232
Uqcrc1	-1.0208864	-0.296073736
Dmac2	-0.9488314	0.444461686
Ndufaf1	-0.9708835	0.03194066
Ndufv1	-1.0221418	-0.566193643
Uqcrc2	-1.1085854	-0.252056039
Atp5g3	-1.1346757	-0.345473009
Ndufab1	-1.1142952	-0.180991265
Lyrm7	-1.124152	-0.021564637
Nubpl	-1.2477358	-0.122705296
Ndufaf4	-0.962453	0.002833335
Ecsit	-1.0924001	-0.19925194
Ndufa9	-1.2446253	-0.261080411
Atp5d	-1.1476006	-0.399153325
Ndufa8	-1.2590306	-0.558061129
Uqcrfs1	-1.3812486	-0.399338964
Cyc1	-1.4582571	-0.391576522
Cox5a	-1.3985978	-0.284874062
Sdhd	-1.4264591	-0.166339546
Sdhaf1	-1.040392	-0.273633988
Uqcc3	-1.1259331	-0.190600272
CYTB	-1.1310548	NA
Ndufaf3	-0.7502474	-0.222054937
Cox17	-0.9665946	0.035717432
Atp5g2	-1.445802	-0.060917272
Cox16	-1.4826865	-0.153852594

Pet100	-1.3660107	0.137595181
ATP8	-1.3624314	NA
Cox14	-1.3947707	-0.250920856
Coa6	-1.3492953	-0.077650056
ND1	-1.5135161	NA
Sdhaf3	-1.5124552	-0.347954829
COX1	-1.5647918	NA
Ndufs3	-1.4618346	-0.206835692
Ndufs7	-1.4305927	-0.424154776
Atp5pb	-1.5268271	NA
Cox4i1	-1.5552002	-0.392573021
Atp5c1	-1.6056417	-0.302613125
Ndufa11	-1.5961987	-0.336168826
Sdhaf4	-1.4323294	-0.416816899
Atp5h	-1.5290384	-0.381397763
Ndufb7	-1.5217255	-0.296442689
Ndufs8	-1.5613788	-0.201742092
Ndufs4	-1.5642756	-0.144984455
Ndufb3	-1.631671	-0.412478422
Cox5b	-1.589757	-0.165038513
Ndufc2	-1.6880588	-0.470595259
Ndufb10	-1.6015473	-0.556267433
Ndufaf6	-1.750717	-0.137017125
Atp5o	-1.7687705	-0.294996224
Ndufb6	-1.7592415	-0.426273328
Sdhb	-1.8498347	-0.348764208
Ndufa12	-1.8038296	-0.271282094
Cox6b1	-1.719599	-0.310830152
Ndufs5	-1.6720756	-0.463137131
Uqcrq	-1.7101388	-0.237861873
Uqcrb	-1.9718259	-0.633399235
Atp5g1	-2.0631913	-0.298894933
Ndufb5	-2.0963976	-0.351905114
Ndufaf5	-2.088548	-0.326547335
Ndufb9	-2.0927709	-0.464815492
Ndufc1	-2.1705367	-0.542889587
Ndufv2	-1.8648816	-0.357439559
Ndufv3	-1.9291854	-0.435076405
Atp5e	-1.8779846	-0.357825694
Ndufb8	-2.0223466	-0.521028625

Uqcr11	-2.0382141	-0.653658507
Atp5k	-2.0702007	-0.235145493
Ndufb2	-1.9584152	-0.632929952
Ndufa5	-1.9966318	-0.579551405
Ndufs6	-1.8890665	-0.338483889
Ndufa13	-1.9430555	-0.439711113
Uqcc2	-1.8967643	-0.624904908
Ndufb11	-1.9847203	-0.419373473
Uqcr10	-1.9493419	-0.505796321
Cox7b	-1.9572527	-0.212457503
Cox7c	-1.9711775	-0.297362105
Uqcrh	-1.9958721	-0.359623176
Ndufa4	-2.0819515	-0.351453062
Ndufb4	-2.0434515	-0.544789848
Atp5mpl	-2.1084523	-0.368939417
Cox6c	-2.0191485	-0.528196793
Atp5l	-2.0087369	-0.503405109
Ndufa2	-2.0531176	-0.352345681
Ndufa6	-2.0879006	-0.335505193
Atp5j2	-2.1339304	-0.36223319
Ndufa3	-1.7960318	-0.097098796
COX2	-1.7838058	NA
ATP6	-1.7189635	NA
Cox20	-1.8557664	-0.339380084
Ndufb1	-1.9691936	NA
COX3	-2.3027704	NA
Atp5md	-2.2387459	-0.291344489
Cox7a1	-2.551529	-1.152111673
ND3	-2.6199258	NA

Table S3. The primer sequences used for this study

Species	Gene	Forward (5′–3′)	Reverse (5′–3′)
Macaca fascicularis	*ARFGAP2*	GCGTCCATCTGAGCTTCATC	CATCATTGGCTGTGCATCCA
	S100A9	CCGGAGGGAATTCAAACAGC	GTTAGCCTCGCCATCAGCAT
	IL1β	GACGTCGATGGCCCTAAACA	GAAGCCCTCGTTGTAGTGCT
Mus musculus	*Casp1*	ACAAGGCACGGGACCTATG	TCCCAGTCAGTCCTGGAAATG
	Ccl2	ACCTGGATCGGAACCAAATG	CCTTAGGGCAGATGCAGTTTTAA
	Ccl7	GCTGCTTTCAGCATCCAAGTG	CCAGGGACACCGACTACTG
	Gapdh (muscle)	TCCCACTCTTCCACCTTCGA	CAGGAAATGAGCTTGACAAAGTTG
	Gapdh (heart)	ATCAACGACCCCTTCATTGACC	CCAGTAGACTCCACGACATACTCAGC
	Gbp2	CTGCACTATGTGACGGAGCTA	GAGTCCACACAAAGGTTGGAAA
	Gbp3	GAGGCACCCATTTGTCTGGT	CCGTCCTGCAAGACGATTCA
	Gbp5	CAGACCTATTTGAACGCCAAAGA	TGCCTTGATTCTATCAGCCTCT
	Gbp7	TCCTGTGTGCCTAGTGGAAAA	CAAGCGGTTCATCAAGTAGGAT
	H2-DMa	CTCGAAGCATCTACACCAGTG	TCCGAGAGCCCTATGTTGGG
	H2-DMb1	ACCCCACAGGACTTCACATAC	GGATACAGCACCCCAAATTCA
	Igtp	CTCATCAGCCCGTGGTCTAAA	CACCGCCTTACCAATATCTTCAA
	Il18	GACTCTTGCGTCAACTTCAAGG	CAGGCTGTCTTTTGTCAACGA
	Il1β	GACGTCGATGGCCCTAAACA	GAAGCCCTCGTTGTAGTGCT
	Il6	TACCACTTCACAAGTCGGAGGC	CTGCAAGTGCATCATCGTTGTTC
	Igf2	CGCTTCAGTTTGTCTGTTCG	AGGTAGACACGTCCCTCTCG
	Irgm1	TGCTCCACTACTCCCCAACAT	GCTCCTACTGACCTCAGGTAAC
	Myd88	TCATGTTCTCCATACCCTTGGT	AAACTGCGAGTGGGGTCAG
	Myh7	ACCAGGCCCTTTGACCTCAAGAAA	TCTTGTCGAACTTGGGTGGGTTCT
	Myog	TACGTCCATCGTGGACAGCAT	TCAGCTAAATTCCCTCGCTGG
	S100a9	ACCACCATCATCGACACCTTC	AAAGGTTGCCAACTGTGCTTC
	Socs1	CTGCGGCTTCTATTGGGGAC	AAAAGGCAGTCGAAGGTCTCG
	Tnf	TTGACCTCAGCGCTGAGTTG	CCTGTAGCCCACGTCGTAGC
	Trim63	TCCTGGACGAGAAGAGAGC	TGCTCCCTGTACTGGAGGAT

Abbreviation: *ARFGAP2*, ADP-ribosylation factor GTPase activating protein 2; *Casp,* caspase 1; *Ccl2*, C-C motif chemokine ligand ; *Ccl7*, C-C motif chemokine ligand 7; *Gapdh*, glyceraldehyde-3-phosphate dehydrogenase; *Gbp2*, guanylate-binding protein 2; *Gbp3*, guanylate-binding protein 3; *Gbp5*, guanylate-binding protein 5; *Gbp7*, guanylate-binding protein 7; *H2-DMa*, histocompatibility 2 class II, locus DMa; *H2-DMb1,* histocompatibility 2 class II, locus Mb1; *Igtp*, interferon gamma-induced GTPase; *Il18*, interleukin 18; *Il1β*, interleukin 1 beta; *Il6*, interleukin 6; *Igf2,* insulin-like growth factor 2; *Irgm1,* immunity-related GTPase family M member 1; *Myd88,* myeloid differentiation primary response gene 88; *Myh7*, myosin heavy chain 7; *Myo,* myogenin; *S100a9*, S100 calcium-binding protein A9; *Socs1*, suppressor of cytokine signaling 1; *Tnf*, tumor necrosis factor alpha; *Trim63*, tripartite motif-containing 63.

Supplemental Reference

1. Castets P, Rion N, Theodore M, Falcetta D, Lin S, Reischl M, et al. mTORC1 and PKB/Akt control the muscle response to denervation by regulating autophagy and HDAC4. Nat Commun. 2019; 10: 3187.
2. Alsaigh T, Evans D, Frankel D, Torkamani A. Decoding the transcriptome of calcified atherosclerotic plaque at single-cell resolution. Commun Biol. 2022; 5: 1084.
3. Henze H, Huttner SS, Koch P, Schuler SC, Groth M, von Eyss B, et al. Denervation alters the secretome of myofibers and thereby affects muscle stem cell lineage progression and functionality. NPJ Regen Med. 2024; 9: 10.
4. Kim S, Jeon JH, Kim M, Lee Y, Hwang YH, Park M, et al. Innate immune responses against mRNA vaccine promote cellular immunity through IFN-beta at the injection site. Nat Commun. 2024; 15: 7226.
5. Butler A, Hoffman P, Smibert P, Papalexi E, Satija R. Integrating single-cell transcriptomic data across different conditions, technologies, and species. Nat Biotechnol. 2018; 36: 411-20.
